# Web-Based Virtual Environment Versus Face-To-Face Delivery for Team-Based Learning of Anesthesia Techniques Among Undergraduate Medical Students: Randomized Controlled Trial

**DOI:** 10.2196/80097

**Published:** 2026-01-15

**Authors:** Darunee Sripadungkul, Suhattaya Boonmak, Monsicha Somjit, Narin Plailaharn, Wimonrat Sriraj, Polpun Boonmak

**Affiliations:** 1 Department of Anesthesiology, Faculty of Medicine Khon Kaen University Muang Khon Kaen, Khon Kaen Thailand

**Keywords:** anesthesia, computer-assisted instruction, distance, education, internet, learning, medical, problem-based learning, students, teaching, virtual reality

## Abstract

**Background:**

Foundational knowledge of anesthesia techniques is essential for medical students. Team-based learning (TBL) improves engagement. Web-based virtual environments (WBVEs) allow many learners to join the same session in real time while being guided by an instructor.

**Objective:**

This study aimed to compare a WBVE with face-to-face (F2F) delivery of the same TBL curriculum in terms of postclass knowledge and learner satisfaction.

**Methods:**

We conducted a randomized, controlled, assessor-blinded trial at a Thai medical school from August 2024 to January 2025. Eligible participants were fifth-year medical students from the Faculty of Medicine, Khon Kaen University, who attended the anesthesiology course at the department of anesthesiology. Students who had previously completed the anesthesiology course or were unable to comply with the study protocol were excluded. They were allocated to one of the groups using a computer-generated sequence, with concealment of allocation to WBVE (on the Spatial platform) or F2F sessions. Both groups received identical 10-section content in a standardized TBL sequence lasting 130 minutes. Only the delivery mode differed (Spatial WBVE vs classroom F2F). The primary outcome was the postclass multiple-choice questionnaire score. The secondary outcome was learner satisfaction. Individual knowledge was assessed before and after the session using a 15-item questionnaire containing multiple-choice questions via Google Forms. Satisfaction was measured immediately after class on a 5-point Likert scale. Outcome scoring and data analysis were blinded to group assignment. Participants and instructors were not blinded.

**Results:**

In total, 79 students were randomized in this study (F2F: n=38, 48%; WBVE: n=41, 52%). We excluded 2% (1/41) of the students in the WBVE group due to incomplete data. There were complete data for the analysis for 78 participants (F2F: n=38, 49%; WBVE: n=40, 51%). Preclass scores were similar between groups (F2F: mean 6.03, SD 2.05; WBVE: mean 6.20, SD 2.04). Postclass knowledge did not differ significantly (F2F: mean 11.24, SD 1.93; WBVE: mean 10.40, SD 2.62; mean difference 0.88, 95% CI –0.18 to 1.94; *P*=.12). Learner satisfaction favored F2F learning across multiple domains, including overall course satisfaction. Overall satisfaction favored F2F learning (mean difference 0.42, 95% CI 0.07-0.77; *P*=.01). Both groups ran as planned. No adverse events were reported. No technical failures occurred in the WBVE group.

**Conclusions:**

In this trial, WBVE-delivered TBL produced similar short-term knowledge gains to F2F delivery, but learner satisfaction was lower in the WBVE group. Unlike many previous studies, this trial compared WBVE and F2F delivery while keeping the TBL curriculum and prespecified outcomes identical across groups. These findings support WBVEs as a scalable option when physical space, learner volume, or constraints are present. However, lower satisfaction in the WBVE highlights the real-world need for improved facilitation, user experience design, and technical readiness before broader implementation.

**Trial Registration:**

Thai Clinical Trials Registry TCTR20240708012; https://www.thaiclinicaltrials.org/show/TCTR20240708012

## Introduction

Foundational knowledge of anesthesia techniques is important for medical students. Medical students are required to have knowledge of anesthesia administration, pharmacology, procedures, complication management, and interpretation of anesthesia records. Traditional didactic lectures offer a clear, efficient way to organize and deliver this content. It also allows broad coverage of key concepts in a limited time. However, limited interactivity can reduce engagement and encourage passive learning [1]. Interactive lectures address these limitations with small-group discussions, problem-solving exercises, and simulations. These activities create a more active classroom and support clinical reasoning. They also improve knowledge retention and provide immediate feedback to guide learning [2,3].

Team-based learning (TBL) is an effective approach to promote collaborative and active learning. TBL links to constructivist learning theory, the interactive-constructive-active-passive framework, and cognitive load theory. A simple sequence includes individual and team readiness assurance, application exercises, and immediate feedback. Students can move beyond just completing tasks to engaging with the content and peers. Additionally, it encourages a deeper understanding of concepts through discussion. Students can focus on what matters, think more deeply, and retain knowledge longer [4-6]. However, TBL also has its limitations. Challenges include managing group dynamics, resolving conflicts arising from differing opinions, managing time effectively, and distributing the workload. It also requires appropriate activity design, resources, and assessments [3,7,8].

Interactive lectures and TBL are used to facilitate the topic of anesthesia techniques for fifth-year medical students in our department. This learning technique demonstrates motivation, problem-based solving, and communication. Advances in medical education technology, such as web-based virtual environments (WBVEs), can support synchronous interaction, shared workspaces, and simulation-like experiences. WBVEs are virtual worlds that enable real-time interactions between users and digital objects through technologies such as virtual and augmented reality. They provide immersive experiences, facilitate collaboration, and support interactive and realistic simulations [9-13]. Compared with conventional TBL, WBVEs may make classes easier to join, easier to expand to more students, and more supportive of group work [7,12,14].

However, comparative evidence remains limited. Existing studies are constrained by heterogeneous reporting, small sample sizes, and nonrandomized designs, particularly in anesthesia education [15-17]. Moreover, learners’ diverse preferences and learning styles should be considered when implementing WBVEs [18]. These limitations justify a randomized trial that isolates delivery mode by comparing WBVE with face-to-face (F2F) instruction under an equivalent TBL design. We conducted a randomized controlled trial comparing WBVE (on the Spatial platform [19]) versus F2F delivery of the same TBL curriculum in anesthesia techniques among fifth-year medical students. Both groups used identical objectives, materials, facilitation, and assessments. Only the delivery mode differed. The primary outcome was postclass knowledge, and the secondary outcome was learner satisfaction. We hypothesized that there would be between-group differences in postclass knowledge and learner satisfaction.

## Methods

### Study Design

This study was a randomized, controlled, single-blinded trial. Double blinding was not feasible as participants were aware of their assigned groups. This study adhered to the CONSORT-EHEALTH (Consolidated Standards of Reporting Trials of Electronic and Mobile Health Applications and Online Telehealth) guidelines [20] to ensure comprehensive and transparent reporting of randomized controlled trial data.

### Participants

We recruited fifth-year medical students from the Faculty of Medicine, Khon Kaen University, who attended the anesthesiology course at the department of anesthesiology. Students who had previously completed the anesthesiology course or were unable to comply with the study protocol were excluded. All participants provided written informed consent. The study was conducted from August 26, 2024, to January 7, 2025. Withdrawal criteria included technical issues, such as internet disruptions or software glitches on the Spatial platform [19].

### Randomization and Recruitment

We used cluster randomization at the class level to minimize contamination between teaching groups. Students were grouped into clusters based on their scheduled learning sessions. Each cluster comprised approximately 20 students. Clusters were then randomly allocated in a 1:1 ratio to either the WBVE or F2F group using computer-generated random numbers. We used a block size of 2 to ensure that each group had a similar number of clusters. The randomization sequence was generated and implemented by investigators who were not involved in teaching or assessment to reduce the risk of allocation bias. For clusters randomized to the WBVE group, students who declined research participation received F2F teaching delivered by another instructor, scheduled at the same time and following an identical teaching plan. For clusters randomized to the F2F group, students who declined research participation attended the same F2F session with the same instructor but were not asked to complete the posttest or satisfaction questionnaire.

### Interventions

#### Preintervention Process

After recruitment into the study, all participants in both groups were given access to the learning material via online learning in Google Classroom, including a slide presentation and supplementary video material, 2 days before the teaching session. On the teaching day, students first received a 5-minute briefing about study procedures and learning objectives. They then completed a questionnaire containing 15 multiple-choice questions (MCQs) before the test in 15 minutes to assess baseline knowledge. Participants also completed a brief questionnaire capturing student characteristics. The test specification table is provided in Multimedia Appendix 1.

#### Session Allocation and Team Setup

After baseline assessments, students attended their assigned session (either a WBVE or an F2F session). In each group, students were organized into 4 TBL teams of 5 to 6 students, supervised by 1 instructor. Each session lasted 130 minutes.

#### Content and Structure Are Common to Both Groups

Both groups participated in a single, standardized 130-minute TBL session. The content was identical, organized into 10 sections on anesthesia techniques (listed in Multimedia Appendix 1). Each section began with core knowledge, followed by a problem-solving scenario aligned with the same learning objectives. Students progressed sequentially through the modules to complete the required tasks.

#### F2F Group

The 10 modules described earlier were delivered F2F using a standard TBL sequence. At the start of the session, the instructor reviewed the learning objectives and ground rules and confirmed team assignments. For each module (Multimedia Appendix 1), students first received a brief minilecture to consolidate core concepts. Subsequently, teams completed a scenario-based application task at their respective tables. They recorded a single team answer. They received immediate feedback, followed by a short whole-class debrief that compared rationales across teams. The room layout comprised 4 separated team tables with a central screen, and teams were asked to discuss quietly to minimize cross talk. Materials were identical to those in the other group (handouts mirroring the slides and identical question stems). Any clarifying questions were addressed at the table before the debrief.

#### WBVE Group

**Figure 1:**
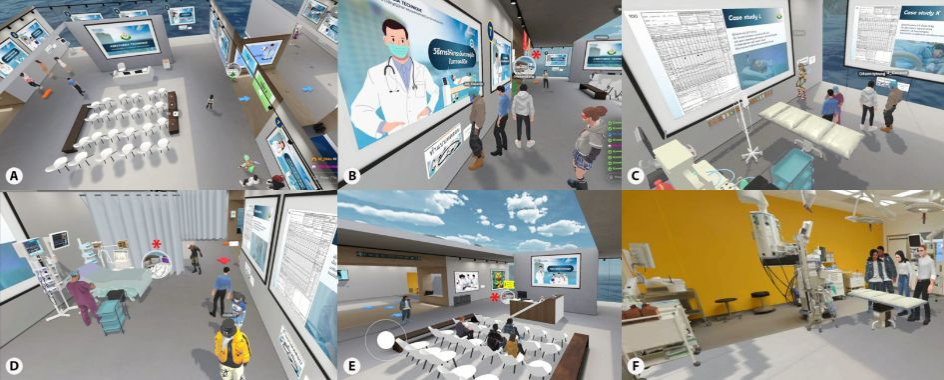
The web-based virtual environment ran on the Spatial platform: (A) A classroom. Students learned modules 1 to 7 in this classroom, organized into 4 teams of 5 to 6 students each, supervised by 1 instructor. (B) A classroom. Students navigated the environment using keyboard-controlled avatars and collaborated in real time via built-in voice and text chat. Students moved to the operating room by clicking the button (*). (C) An operating room. Students learned modules 8 and 9 in this room, including how to read anesthetic record data. Each group also discussed 4 case studies for team-based learning. (D) An operating room. After finishing the questions, students moved to the common room by clicking the button (*). (E) A common room. Students in each group asked clarifying questions in this room, after which the instructor conducted a debriefing for module 10. If students wanted to review the material again or go to an operating room or a classroom, they could return by clicking the button (*). (F) A common room. A simulated operating room had been created to allow students to learn in a realistic virtual environment before entering the actual operating room. If students had extra time while waiting for their classmates, they could explore this room. Various games were also available to help train their minds.

Sessions ran on the Spatial platform (Spatial Systems, Inc; Thai localization) [19] using desktop computers on the university’s secure local network. On first log-in, students created an account and entered a virtual space with 3 areas (a classroom, an operating room, and a common room). Each was equipped with detailed 3D anesthesia equipment to support the team’s application tasks. The same 10 interactive modules described earlier were delivered in sequence. Each module began with brief core content, followed by a scenario-based application task aligned with the learning objectives. Students moved through the environment with keyboard-controlled avatars and collaborated in real time via built-in voice and text chat. Embedded multimedia (short videos, slide decks, interactive cases, and simple educational games) supported engagement. The instructor monitored team rooms, offered real-time guidance, provided immediate feedback, and answered questions as they arose. Any clarifying questions were addressed in the common room before the debrief. A detailed description of the WBVE is provided in Figure 1.

#### Postintervention Process

Immediately after the teaching session, 15 minutes were allocated for outcome measurement. All students completed a questionnaire containing 15 MCQs after the test via Google Forms, using the exact test specification as before the test, under invigilated conditions. They then completed a satisfaction questionnaire in Google Forms (5-point Likert scale; instrument and scoring details are provided in Multimedia Appendix 1). The forms were identical for both groups and were accessed through locked links. Answer keys were withheld until all submissions had been received. In addition, we enabled automatic scoring and time stamp logging, restricted responses to a single submission per account, and exported raw data directly from Google Forms into the analysis dataset.

### Data Collection

Pre- and postintervention knowledge evaluations were assessed using a 15-item MCQ test mapped to a test specification table (Multimedia Appendix 1). The authors designed and developed a questionnaire on students’ satisfaction with the learning process, drawing on previous studies [21,22], and adjusted its content for use in a Thai context (Multimedia Appendix 1). Student satisfaction was evaluated immediately after the intervention using a 21-item survey, with responses on a 5-point Likert scale (1=strongly disagree, 2=disagree, 3=neither agree nor disagree, 4=agree, and 5=strongly agree). The questionnaire’s content validity was confirmed by 3 experts. Internal consistency was excellent, with a Cronbach α of 0.95. We also recorded gender, age, grade point average, and experience with WBVEs (including frequency, proficiency, and comfort).

### Statistical Analysis

Data were analyzed using Stata/SE (version 18.0; StataCorp) for Windows. Descriptive statistics summarized participant characteristics. Categorical variables were presented as counts and percentages, and continuous variables were presented as means and SDs. Proportions were calculated using nonmissing denominators. Analyses followed the intention-to-treat principle. Because only 1 (1%) of the 79 participants had missing primary outcome data, analyses were conducted using complete-case analysis. Missing completely at random testing and multiple imputation were not performed due to negligible missingness. Between-group comparisons were conducted using linear mixed modeling. We reported the mean differences and 95% CIs. For the primary outcome (knowledge), we used posttest score as the dependent variable, study group as the factor, and pretest score as a covariate. For multiple tests that were analyzed, the false discovery rate was controlled at 5% (Benjamini-Hochberg method). Item-level Likert responses were summarized descriptively as means (SDs).

The required sample size was calculated using the postlearning knowledge score from 63 fifth-year medical students who studied the anesthesia technique in the previous academic year (2023), with a mean of 57.94% (SD 14.07). To detect a 10% difference in knowledge score (type I error of 0.05) with 80% power, we determined that a sample size of 32 participants in each group would be required. The dropout rate was accounted for in the sample size, which included at least 40 participants per group, representing approximately 10% of the total dropout rate.

### Ethical Considerations

This study received full board approval from the human research ethics committee of Khon Kaen University (HE671294). It was also registered with the Thai Clinical Trials Registry before participant enrollment (TCTR20240708012). This study was conducted in accordance with the World Medical Association Declaration of Helsinki and institutional policies. Participants received clear written information about the study objectives, procedures, potential risks, and data management before participating. Participation was voluntary, and they could withdraw at any time without affecting their academic standing. Those who agreed to take part then provided written informed consent. Study-related data were not collected from them. After participating in this study, students created unique codes instead of using their names, which enabled us to link pre- and posttest data without knowing individual identities to protect privacy and confidentiality. Data were anonymized for analysis and stored on password-protected systems with access restricted to the investigator’s team. Students did not receive any financial or in-kind compensation.

## Results

### Overview

**Figure 2:**
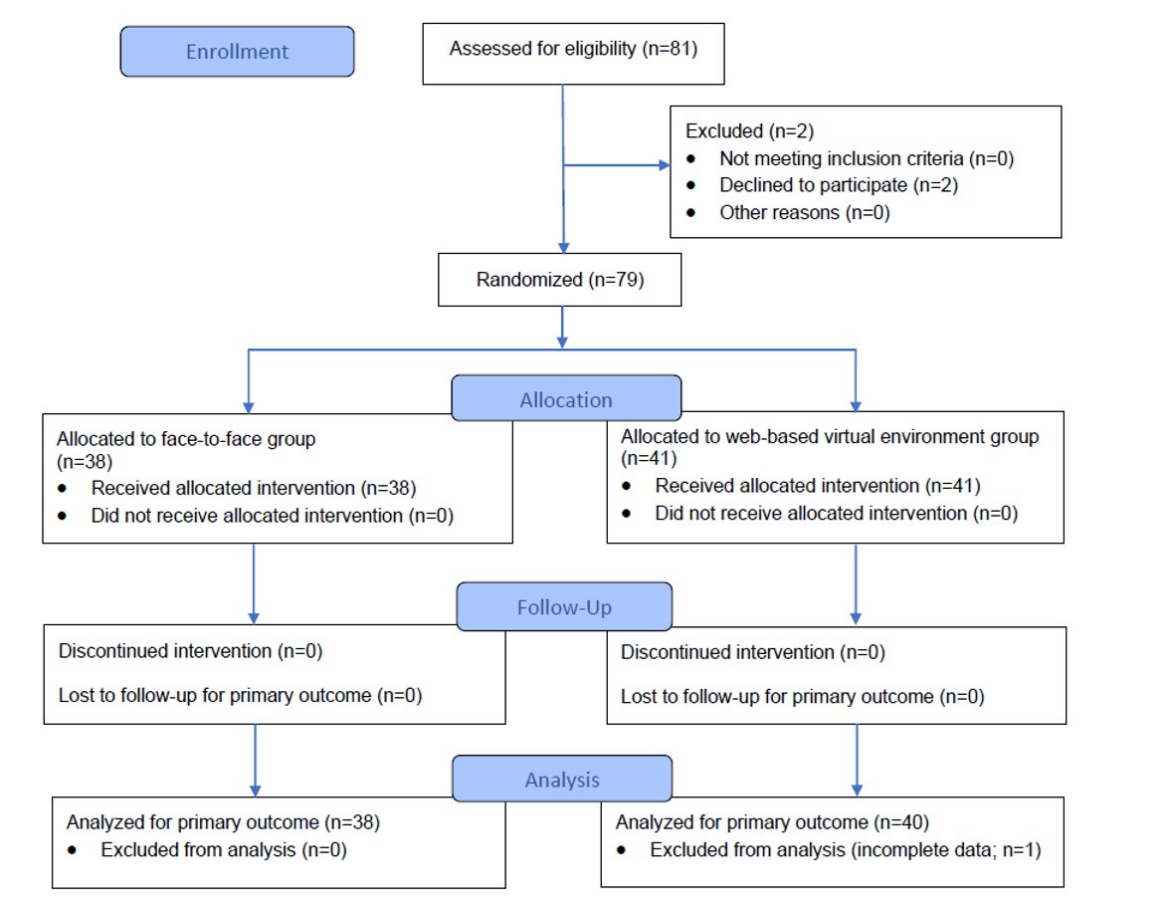
Flow diagram of the participants in the study.

A total of 81 participants were assessed for eligibility; 2 (2%) participants declined to participate. Both were in clusters randomized to the F2F group; therefore, they received F2F teaching as part of the regular course. However, their data were not included in the analysis. Hence, 79 participants were enrolled in the study and allocated into 2 groups: the F2F group (n=38, 48%) and the WBVE group (n=41, 52%); 2% (1/41) of the participants in the WBVE group had incomplete outcome data and were excluded. A total of 78 (99%) participants had complete data in the analysis (Figure 2).

### Participant Characteristics

As shown in Table 1, 78 participants were included, with 38 (49%) assigned to the F2F group and 40 (51%) to the WBVE group. The groups were comparable in gender, age, and grade point average. The proportion of male participants was slightly higher in the F2F group (25/38, 66%) compared to the WBVE group (21/40, 52%). Most participants in both groups reported limited previous exposure to WBVEs, with more than 60% (51/78) of the participants classified as novices and a few reporting intermediate or higher proficiency. Comfort levels with the technology were predominantly neutral.

**Table 1:** Baseline characteristics by group in a cluster-randomized trial of a face-to-face (F2F) group versus a web-based virtual environment (WBVE) group for team-based learning among fifth-year medical students (N=78).

				F2F group (n=38)		WBVE group (n=40)
Male, n (%)				25 (66)		21 (52)
Age (y), mean (SD)				22.5 (0.6)		22.5 (0.7)
Grade point average (0-4), mean (SD)				3.50 (0.34)		3.44 (0.33)
**Experience with WBVEs, n (%)**						
	**Frequency**					
		No	19 (50)		26 (65)	
		Sometimes	18 (47)		14 (35)	
		Frequently	1 (3)		0 (0)	
	**Proficiency**					
		Novice	24 (63)		27 (67)	
		Beginner	10 (26)		7 (17)	
		Intermediate	4 (11)		5 (13)	
		Advanced	0 (0)		1 (3)	
		Expert	0 (0)		0 (0)	
	**Comfort level**					
		Discomfort	0 (0)		2 (5)	
		Neutral	27 (71)		27 (67)	
		Comfort	11 (29)		11 (28)	

### Knowledge Outcomes

As shown in Table 2, both groups demonstrated significant improvement in knowledge after the intervention. The WBVE group had slightly higher mean pretest scores than the F2F group (mean 6.20, SD 2.04 vs mean 6.03, SD 2.05 for WBVE vs F2F), but lower posttest scores (mean 10.40, SD 2.62 vs mean 11.24, SD 1.93 for WBVE vs F2F). However, after adjustment using linear mixed modeling and the Benjamini-Hochberg procedure, no statistically significant differences between groups were observed in either pretest or posttest scores (*P*=.70 and *P*=.12, respectively).

**Table 2:** Knowledge (15-point scale) and learner satisfaction (Likert 1-5) by group in a cluster-randomized trial comparing face-to-face (F2F) versus web-based virtual environment (WBVE) team-based learning among fifth-year medical students (N=78).

						F2F group (n=38), mean (SD)			WBVE group (n=40), mean (SD)			Mean difference (95% CI)			*P* value
**Knowledge (15-point scale)^a^**															
	Pretest knowledge score				6.03 (2.05)			6.20 (2.04)			–0.17 (–1.07 to 0.72)			.70	
	Posttest knowledge score				11.24 (1.93)			10.40 (2.62)			0.88 (–0.18 to 1.94)			.12	
**Learner satisfaction (Likert scale 1-5)^b^**															
	**Learning topic**														
		Intellectually challenging and stimulating		4.7 (0.5)			4.4 (0.6)			0.34 (0.05 to 0.62)			.02^c^		
		Valuable learning gained		4.9 (0.3)			4.6 (0.6)			0.29 (0.04 to 0.54)			.02^c^		
		Increased interest in the subject		4.6 (0.7)			4.5 (0.7)			0.10 (–0.21 to 0.41)			.53		
	**Learning process**														
		**Instructor**													
			Instructor enthusiasm	4.9 (0.3)			4.7 (0.6)			0.22 (–0.01 to 0.45)			.07		
			Presentation style holds interest	4.8 (0.6)			4.4 (0.9)			0.36 (–0.01 to 0.74)			.06		
			Clear explanations	4.8 (0.5)			4.3 (0.8)			0.46 (0.08 to 0.85)			.01^c^		
			Facilitates note-taking	4.6 (0.7)			4.4 (1.0)			0.28 (–0.11 to 0.67)			.17		
			Friendly to students	4.9 (0.2)			4.9 (0.4)			0.10 (–0.06 to 0.25)			.25		
			Welcomes questions or help	4.9 (0.3)			4.8 (0.5)			0.12 (–0.07 to 0.31)			.25		
			Genuine interest in students	4.9 (0.3)			4.8 (0.5)			0.07 (–0.10 to 0.24)			.46		
			Presents alternative viewpoints	4.8 (0.4)			4.6 (0.6)			0.22 (–0.04 to 0.48)			.11		
		**Technique**													
			Well-prepared materials	4.8 (0.4)			4.3 (1.0)			0.49 (0.08 to 0.90)			.01^c^		
			Encourages class discussion	4.9 (0.4)			4.1 (1.0)			0.77 (0.27 to 1.27)			<.001^c^		
			Invites the sharing of ideas	4.8 (0.4)			3.9 (1.1)			0.92 (0.34 to 1.50)			<.001^c^		
			Encourages and answers questions	4.9 (0.4)			4.6 (0.7)			0.32 (0.04 to 0.60)			.02^c^		
			Supports student expression	4.9 (0.3)			4.5 (0.8)			0.42 (0.07 to 0.76)			.009^c^		
			Technical satisfaction (audio, video, and media)	4.7 (0.5)			4.4 (0.8)			0.28 (–0.06 to 0.62)			.11		
			Satisfaction with teaching methods	4.9 (0.3)			4.3 (0.9)			0.54 (0.12 to 0.97)			.004^c^		
	**Learning outcome**														
		Content covers objectives		4.9 (0.3)			4.7 (0.5)			0.27 (0.05 to 0.50)			.01^c^		
		Consistent with current knowledge		4.9 (0.4)			4.7 (0.5)			0.22 (–0.02 to 0.45)			.07		
	Overall course satisfaction				4.9 (0.4)			4.5 (0.8)			0.42 (0.07 to 0.77)			.01^c^	

^a^Knowledge scores were on a 15-point scale (0-15); higher scores indicated greater knowledge.

^b^Likert score (1=very dissatisfied, 2=dissatisfied, 3=neither satisfied nor dissatisfied, 4=satisfied, and 5=very satisfied).

^c^Statistically significant difference at *P*<.05.

### Learner Satisfaction

As shown in Table 2, learner satisfaction across multiple domains was generally high in both groups. However, several aspects favored the F2F group. For the learning topic, the F2F group reported higher levels of intellectual challenge and stimulation (mean difference 0.34; 95% CI 0.05-0.62; *P*=.02) as well as greater perceived value in learning gained (mean difference 0.29; 95% CI 0.04-0.54; *P*=.02). For the learning process, the evaluation was divided into instructor- and technique-related components. The F2F group rated significantly higher on clear explanations (mean difference 0.46; 95% CI 0.08-0.85; *P*=.01) on instructor-related items. It also rated significantly higher on well-prepared materials (mean difference 0.49; 95% CI 0.08-0.90; *P*=.01), encouragement of class discussion (mean difference 0.77; 95% CI 0.27-1.27; *P<*.001), an invitation to share ideas (mean difference 0.92; 95% CI 0.34-1.50; *P<*.001), encouragement and answering questions (mean difference 0.32; 95% CI 0.04-0.60; *P=*.02), support for student expression (mean difference 0.42; 95% CI 0.07-0.76; *P=*.009), and satisfaction with teaching methods (mean difference 0.54; 95% CI 0.12-0.97; *P=*.004). In terms of learning outcomes, the F2F group rated significantly higher on content coverage (mean difference 0.27; 95% CI 0.05-0.50; *P*=.01). While overall course satisfaction was significantly higher in the F2F group mean 4.9, SD 0.4 vs mean 4.5, SD 0.8; mean difference 0.42; 95% CI 0.07-0.77; *P*=.01.

### Harms

Neither group experienced adverse events, privacy breaches, or unintended effects. The WBVE session ran as planned, and no technical issues (audio or latency) were observed. There were no withdrawals due to technical problems.

## Discussion

### Principal Findings

This study compared knowledge and learner satisfaction between F2F and WBVE learning for anesthesia techniques. Both groups were comparable in terms of characteristics, including their experience with WBVEs. All participants showed improvement in knowledge outcomes. However, posttest scores did not show statistically significant differences between groups. Learner satisfaction was generally high in both groups, while it was consistently higher in the F2F group. The F2F group reported significantly higher satisfaction with the learning topic, the learning process (related to learning techniques), and the learning outcome. Overall course satisfaction was higher in the F2F group.

### Participant Characteristics

The baseline characteristics of participants in both groups were comparable. There were no significant differences in gender, age, or academic performance. Most participants in both groups reported limited previous experience with WBVEs, with more than 60% (51/78) of the participants classified as novices. Most (54/78, 69%) participants reported neutral comfort ratings. This suggests that students were not very confident with WBVEs but were not uncomfortable with them. This limited exposure may have influenced both their comfort level and engagement with the WBVEs [23,24]. Previous studies have reported that WBVEs are not an obstacle to learning for medical students with limited experience with them [23-25]. The findings indicate that additional training and familiarization sessions with WBVEs may be necessary to enhance learner confidence and optimize engagement [23-25].

### Knowledge Outcome

Pre- and posttest results showed improvement in both groups. Posttest scores also did not show statistically significant differences between groups. This suggests that both the WBVE and F2F delivery are effective for teaching. This is consistent with previous studies demonstrating the potential of WBVEs to facilitate cognitive learning and performance in medical students [25-29]. Despite technical and practical challenges, the use of WBVEs can improve knowledge and performance [25-29].

### Learner Satisfaction

Our data reported that overall satisfaction was high in both groups. However, across multiple domains of learner satisfaction, the F2F instructional modality was favored. The F2F group reported significantly higher ratings for intellectual stimulation and for the value of what they learned. They also felt that instructors provided clearer explanations and more effectively encouraged idea sharing, questioning, answering, and student expression. Our data showed the benefit of the F2F format in promoting effective engagement, interactive learning dynamics, and real-time interpersonal communication. These findings suggest that conducting TBL in WBVEs is faced with several limitations. Technical constraints and students’ limited familiarity with WBVE may affect learner satisfaction [3,23,24]. Additionally, the instructor’s facilitation techniques during WBVE should be a concern [23,24].

Cultural context may help explain the differences in satisfaction [29-32]. However, it should be viewed as a possible moderator rather than a fixed or deterministic cause. Thai medical students may defer to instructors and be comfortable with structured guidance and supervision. This may favor F2F settings, where nonverbal cues and immediate instructor feedback are more noticeable. Consistent with previous work in Asia, students have reported greater satisfaction with traditional methods [30]. This appears to be influenced by personality, self-efficacy, and their expectations about instructional design and delivery [30]. In contrast, studies and systematic reviews from Europe and the United States have often found higher satisfaction with WBVEs [29,31]. However, cultural context does not uniformly predict outcomes. Our data and previous studies observed variability across participants that appears to depend on facilitation quality, previous exposure, technology self-efficacy, language demands, and device access [29-32]. During the COVID-19 pandemic, Thai medical students adapted quickly [32]. They reported greater satisfaction with new anesthesiology learning technologies than their teachers. These suggest that growing familiarity can narrow culture-based gaps over time [32]. We view cultural context as one of several interacting factors that affect learner satisfaction.

The variability in learner satisfaction likely reflects implementation issues rather than only the delivery method [29-32]. In WBVEs, instructors cannot rely on being physically in the room, so they must think more carefully about how they facilitate the learning process [10,12,29]. It is essential to use strategies that build social presence and keep students engaged [10,12,29]. Additionally, technical issues, such as latency, audio problems, and complex navigation, can create more burdens [10,12]. When the sound is delayed, the flow of discussion is broken. It becomes hard to know when to speak or respond [10,12]. Complex navigation may be engaging at first, but students must spend time figuring out how to move between virtual rooms or use the interface. They end up focusing on the platform rather than the TBL tasks and the content itself [2,10,12]. Students’ satisfaction is likely to depend on how well learning is facilitated [10,12]. Reliable devices and technical support should be included [10,12]. Therefore, future work should concentrate on strengthening facilitation and improving the user experience.

### Implications

Our findings indicate that WBVEs can yield knowledge gains comparable to those of F2F instruction. WBVEs may be especially valuable in settings with limited classroom space, tight schedules, or large cohorts [10,12,16]. However, running TBL in WBVEs requires careful design and facilitation to keep students engaged and improve their satisfaction [2,10,12,29]. Specifically, this includes clearer turn taking, more visible feedback, simpler navigation within the virtual environment, and fewer technical obstacles [2,10,12,29]. This study’s main contribution is to separate delivery mode from pedagogy. We directly compared a WBVE with F2F delivery under an identical TBL design using assessor-blinded, prespecified outcomes. These design features extend previous work, which often combined different instructional methods or did not report use and outcome measures as clearly [15-17]. This approach can be implemented with medical students, especially in anesthesiology education.

### Limitations

This study has several limitations. A single-center study may limit the generalizability of the findings. The relatively small sample size may limit their applicability to broader and more diverse student populations. Next, a double-masked study was not feasible due to the nature of the intervention. This may have introduced performance or response bias. Additionally, participants had limited previous exposure to WBVEs. This may have dampened their engagement and satisfaction. Cultural factors specific to Thai medical students should be a concern. A preference for structured, instructor-led learning may have contributed to the lower satisfaction reported with WBVE.

### Future Directions

Future research should include larger sample sizes and multicenter trials. The generalizability of the findings across diverse learner populations should be of concern. In addition, future studies should examine how students’ familiarity with WBVEs affects their satisfaction and learning outcomes. They should test whether repeated exposure helps students feel more comfortable and engaged. Moreover, researchers should focus on facilitation techniques to identify the most effective approach for WBVE-based TBL. Simulation-based features within WBVEs can make the environment more interactive and better aligned with students’ learning needs. Further work should also study instructional designs tailored to Asian medical education. These designs should incorporate strategies that support collaborative learning and respect cultural norms.

### Prior Presentation

Part of this study was previously presented as an oral presentation at the SANCON-ASPA 2025 conference—Scaling New Heights in Pediatric Anesthesia and Beyond (24th Annual Conference of the Society of Anesthesiologists of Nepal [SANCON] and 21st Meeting of the Asian Society of Pediatric Anesthesiologists [ASPA]), held in Kathmandu, Nepal, on April 5, 2025. The abstract was presented under the title “Metaverse versus in-person: impact on anesthesia education and student satisfaction.”

### Conclusions

This trial is innovative in isolating the delivery mode from pedagogy by directly comparing WBVE to F2F delivery within an identical TBL structure with prespecified end points. This design differs from much of the existing literature, in which shifts in instructional strategy often accompany technological changes. It is challenging to attribute effects solely to the delivery mode. Our findings contribute evidence that a WBVE can deliver comparable short-term knowledge gains in a single standardized session. It has practical implications for scaling instruction when classroom space, faculty time, or scheduling is limited. Nevertheless, lower satisfaction in WBVE underscores the need for real-world implementation. We require stronger facilitation strategies, improved social presence, simplified navigation, and robust technical support to achieve an experience comparable to F2F.

## Appendix

Multimedia Appendix 1 Instructional package: lesson plan, user guide, and assessment instruments.

Multimedia Appendix 2 CONSORT-eHEALTH (V 1.6.1).

## Abbreviations

CONSORT-EHEALTH: Consolidated Standards of Reporting Trials of Electronic and Mobile Health Applications and Online Telehealth
F2F: face-to-face
MCQ: multiple-choice question
TBL: team-based learning
WBVE: web-based virtual environment

## Data Availability

The datasets generated or analyzed during this study are available from the corresponding author on reasonable request.
