# Supplementary material for: Web-Based Virtual Environment Versus Face-To-Face Delivery for Team-Based Learning of Anesthesia Techniques Among Undergraduate Medical Students: Randomized Controlled Trial
Source: JMIR Med Educ. 2026 Jan 15;12:e80097. doi: 10.2196/80097 (PMC12856400; doi:10.2196/80097)
Supplement: Multimedia Appendix 1 [file mededu_v12i1e80097_app1.docx]

**Instructional Package: Lesson Plan, User Guide, and Assessment Instruments**

1. Lesson plan
2. User Guide for Spatial
3. Specification table for Multiple-Choice Questions
4. Medical Student Satisfaction Questionnaire on Learning Process
5. **Lesson plan**

**Anesthesia techniques – learning objective**

The objectives of this topic include understanding responses to surgical stimuli, defining anesthesia, recognizing the benefits of anesthesia, identifying levels of anesthesia, understanding standard care in anesthesia, familiarizing with anesthetic agents including intravenous anesthetic drugs, inhalation anesthetic drugs, muscle relaxants (including mechanisms, actions, and doses), assessing benefits and risks, comprehending types and concepts of each anesthesia technique, recognizing complications associated with each anesthesia technique, interpreting anesthetic records, and case study (interpret anesthetic record, discuss risks and benefits of anesthesia for each patient).

Teaching content was aligned with the existing anesthesia curriculum and reviewed by the anesthesiology faculty. Quality was ensured mainly via content and instrument validation.

**F2F delivery TBL lesson plan**

- Introduction to anesthesia technique 2 minutes
- Response to surgical stimuli 2 minutes
- Anesthesia definition and the benefit of anesthesia 2 minutes
- Level of anesthesia and standard care for 15 minutes
- Anesthetic agent (intravenous anesthetic drugs, inhalation anesthetic drugs, muscle relaxants): mechanism, action, dose, benefits, and risks 45 minutes
- Anesthesia technique (concept and type) 15 minutes
- Complication 5 minutes
- Anesthesia records 9 minutes
- Case study (interpret anesthetic record, discuss risks and benefits of anesthesia for each patient): 30 minutes
- Conclusion 5 minutes
- During the final 15 minutes of the class
- The instructor summarizes all of the content covered by asking the medical students to summarize according to the following points jointly:
  - Explain the principles and benefits of anesthesia.
  - Describe the different levels of anesthesia/sedation (minimal sedation, moderate sedation, deep sedation, general anesthesia).
  - Explain the pharmacology of drugs used for anesthesia, including intravenous agents, inhalation agents, and muscle relaxants.
  - Classify the clinical techniques for administering anesthesia and describe the perioperative patient management (TIVA, balanced general anesthesia, inhalational general anesthesia, VIMA).
  - Identify complications arising from the administration of anesthetic drugs.
- Posttest (15 MCQs with Google form)

**WBVE delivery TBL lesson plan**

- This Spatial composed of two rooms. One is a basic concept room, and the other is an operations room (with four small rooms inside).
- Each team must complete the questions to achieve the case-solving aim for each learning content.
- The only route to encounter the content of each topic sequentially.
- Each person will have a login and either a laptop or a tablet. It is advisable to have headphones.
- Travel together in groups, but with four separate routes and identical content for each group.
- Assistance is available through the ability to ask the teacher questions when needed.
- The content is divided into ten segments, and there will be question-answering sessions where each person has their answer but works together. Aggregate group scores from the questions answered.
- Content 1: Introduction to anesthesia technique 2 minutes (VDO-narrative presentation) + Response to surgical stimuli 2 minutes (cartoon) (VDO-narrative presentation) + Anesthesia definition and benefit of anesthesia 2 minutes (VDO-narrative presentation)
- Content 2: Level of anesthesia and standard care 15 minutes (VDO-narrative presentation, 8 MCQs)
- Content 3: Anesthetic agent (intravenous anesthetic drugs): mechanism, action, dose, benefits, and risks 15 minutes (VDO-narrative presentation, 2 MCQs)
- Content 4: Anesthetic agent (inhalation anesthetic drugs): mechanism, action, dose, benefits, and risks 15 minutes (VDO-narrative presentation, 2 MCQs)
- Content 5: Anesthetic agent (muscle relaxants): mechanism, action, dose, benefits, and risks 15 minutes (VDO-narrative presentation, 2 MCQs)
- Content 6: Anesthesia technique (concept and type) 15 minutes (VDO-narrative presentation, 4 MCQs)
- Content 7: Complication 5 minutes (VDO-narrative presentation)
- Content 8: Anesthesia records 9 minutes (VDO-narrative presentation)
- Content 9 (4 case studies, I, J, K, and L): How to interpret the anesthetic record, go to four operating rooms, see the anesthetic record, answer the questions, 30 minutes (12 MCQs)
- Content 10: Take-home message, conclusion for every topic, 5 minutes
- During the final 15 minutes of the class
- The instructor summarizes all of the content covered by asking the medical students to summarize according to the following points jointly:
  - Explain the principles and benefits of anesthesia.
  - Describe the different levels of anesthesia/sedation (minimal sedation, moderate sedation, deep sedation, general anesthesia).
  - Explain the pharmacology of drugs used for anesthesia, including intravenous agents, inhalation agents, and muscle relaxants.
  - Classify the clinical techniques for administering anesthesia and describe the perioperative patient management (TIVA, balanced general anesthesia, inhalational general anesthesia, VIMA).
  - Identify complications arising from the administration of anesthetic drugs.
- Posttest (15 MCQs with Google form)
- No major revisions to the WBVE occurred during August 2024–January 2025; the system and materials were frozen for the trial.
- WBVE group did not experience any technical problems.

1. **User Guide for Spatial**

1. **Specification table for Multiple-Choice Questions**

A total of 15 four-choice multiple-choice questions (MCQs) were developed for both pre-and post-intervention knowledge evaluations. The content of the MCQs covers key aspects of anesthesia, distributed across various subtopics as follows:

- One question assesses the ability to recognize the benefits of anesthesia.
- One question evaluates knowledge in identifying the different levels of anesthesia.
- One question focuses on understanding standard anesthesia care practices.
- One question addresses the pharmacology of intravenous anesthetic agents.
- Two questions cover the clinical application of intravenous anesthetics.
- One question examines the pharmacology of inhalation anesthetic agents.
- Two questions assess the clinical application of inhalation anesthetics.
- One question tests knowledge of the pharmacology of muscle relaxants.
- Two questions focus on the clinical application of muscle relaxants.
- Two questions evaluate understanding of the types and concepts of various anesthesia techniques.
- One question addresses complications associated with anesthesia techniques.

1. **Medical Student Satisfaction Questionnaire on Learning Process**

*Likert scale: 1 (Very dissatisfied), 2 (Dissatisfied), 3 (Neither satisfied nor dissatisfied), 4 (Satisfied), 5 (Very satisfied)*

I. Learning Topics

- I find the course intellectually challenging and stimulating.
- I have learned something that I consider valuable.
- My interest in the subject has increased significantly due to this course.

II. Learning Process

Instructor

- The instructor is enthusiastic about teaching the course.
- The instructor's presentation style holds my interest during class.
- The instructor's explanations are clear.
- The instructor gives lectures that facilitate note-taking.
- The instructor is friendly towards individual students.
- The instructor makes students feel welcome when seeking help or advice, both in and outside of class.
- The instructor has a genuine interest in individual students.
- The instructor presents points of view other than their own when appropriate.

Teaching Techniques

- The course materials are well-prepared and carefully explained.
- Students are encouraged to participate in class discussions.
- Students are invited to share their ideas and knowledge.
- Students are encouraged to ask questions and receive meaningful answers.
- Students are encouraged to express their ideas and ask questions of the instructor.
- Satisfaction with technical aspects (audio, video, media).
- Satisfaction with teaching methods.

III. Learning Outcomes

- The content covers the stated objectives and necessary knowledge.
- The content is appropriately aligned with current knowledge.

IV. Overall Course Satisfaction
