# Supplementary material for: Web-Based Virtual Environment Versus Face-To-Face Delivery for Team-Based Learning of Anesthesia Techniques Among Undergraduate Medical Students: Randomized Controlled Trial
Source: JMIR Med Educ. 2026 Jan 15;12:e80097. doi: 10.2196/80097 (PMC12856400; doi:10.2196/80097)
Supplement: Multimedia Appendix 2 [file mededu_v12i1e80097_app2.pdf]

CONSORT-EHEALTH (V 1.6.1) - Submission/Publication Form

The CONSORT-EHEALTH checklist is intended for authors of randomized trials evaluating web-based and Internet-based applications/interventions, including mobile interventions, electronic games (incl multiplayer games), social media, certain telehealth applications, and other interactive and/or networked electronic applications. Some of the items (e.g. all subitems under item 5 - description of the intervention) may also be applicable for other study designs.

The goal of the CONSORT EHEALTH checklist and guideline is to be
a) a guide for reporting for authors of RCTs,
b) to form a basis for appraisal of an ehealth trial (in terms of validity)

CONSORT-EHEALTH items/subitems are MANDATORY reporting items for studies published in the Journal of Medical Internet Research and other journals / scientific societies endorsing the checklist.

Items numbered 1., 2., 3., 4a., 4b etc are original CONSORT or CONSORT-NPT (non-pharmacologic treatment) items.
Items with Roman numerals (i., ii, iii, iv etc.) are CONSORT-EHEALTH extensions/clarifications.

As the CONSORT-EHEALTH checklist is still considered in a formative stage, we would ask that you also RATE ON A SCALE OF 1-5 how important/useful you feel each item is FOR THE PURPOSE OF THE CHECKLIST and reporting guideline (optional).

Mandatory reporting items are marked with a red *.
In the textboxes, either copy & paste the relevant sections from your manuscript into this form - please include any quotes from your manuscript in QUOTATION MARKS,
or answer directly by providing additional information not in the manuscript, or elaborating on why the item was not relevant for this study.

YOUR ANSWERS WILL BE PUBLISHED AS A SUPPLEMENTARY FILE TO YOUR PUBLICATION IN JMIR AND ARE CONSIDERED PART OF YOUR PUBLICATION (IF ACCEPTED).
Please fill in these questions diligently. Information will not be copyedited, so please use proper spelling and grammar, use correct capitalization, and avoid abbreviations.

DO NOT FORGET TO SAVE AS PDF _AND_ CLICK THE SUBMIT BUTTON SO YOUR ANSWERS ARE IN OUR DATABASE !!!

Citation Suggestion (if you append the pdf as Appendix we suggest to cite this paper in the caption):
Eysenbach G, CONSORT-EHEALTH Group

CONSORT-EHEALTH: Improving and Standardizing Evaluation Reports of Web-based and Mobile Health Interventions
J Med Internet Res 2011;13(4):e126
URL: http://www.jmir.org/2011/4/e126/
doi: 10.2196/jmir.1923
PMID: 22209829

bpolpu@kku.ac.th Switch account

Draft not saved

Not shared

* Indicates required question

Your name *

First Last

Suhattaya Boonmak

Primary Affiliation (short), City, Country *

University of Toronto, Toronto, Canada

Khon Kaen University, Khon Kaen, Thailand

Your e-mail address *

abc@gmail.com

suhabo@kku.ac.th

Title of your manuscript *

Provide the (draft) title of your manuscript.

Web-Based Virtual Environment Versus Face-to-Face Delivery of Team-Based Learning for Anesthesia Techniques in Undergraduate Medical Students: A Randomized Controlled Trial

Name of your App/Software/Intervention *

If there is a short and a long/alternate name, write the short name first and add the long name in brackets.

Spatial [Web-Based Virtual Environment]

Evaluated Version (if any)

e.g. "V1", "Release 2017-03-01", "Version 2.0.27913"

V1 18112025

Language(s) *

What language is the intervention/app in? If multiple languages are available, separate by comma (e.g. "English, French")

Thai language

URL of your Intervention Website or App

e.g. a direct link to the mobile app on app in appstore (itunes, Google Play), or URL of the website. If the intervention is a DVD or hardware, you can also link to an Amazon page.

https://www.spatial.io

URL of an image/screenshot (optional)

https://www.spatial.io

Accessibility *

Can an enduser access the intervention presently?

- ○ access is free and open
- ◉ access only for special usergroups, not open
- ○ access is open to everyone, but requires payment/subscription/in-app purchases
- ○ app/intervention no longer accessible
- ○ Other: ______

Primary Medical Indication/Disease/Condition *

e.g. "Stress", "Diabetes", or define the target group in brackets after the condition, e.g. "Autism (Parents of children with)", "Alzheimers (Informal Caregivers of)"

Learning the anesthesia techniques topic in Ar

Primary Outcomes measured in trial *

comma-separated list of primary outcomes reported in the trial

Knowledge, Satisfaction

Secondary/other outcomes

Are there any other outcomes the intervention is expected to affect?

No, there are not any other outcomes the intervention is expected to affect.

Recommended "Dose" *

What do the instructions for users say on how often the app should be used?

- ( ) Approximately Daily
- ( ) Approximately Weekly
- ( ) Approximately Monthly
- ( ) Approximately Yearly
- (x) "as needed"
- ( ) Other:

Approx. Percentage of Users (starters) still using the app as recommended after 3 months *

- [x] unknown / not evaluated
- [ ] 0-10%
- [ ] 11-20%
- [ ] 21-30%
- [ ] 31-40%
- [ ] 41-50%
- [ ] 51-60%
- [ ] 61-70%
- [ ] 71%-80%
- [ ] 81-90%
- [ ] 91-100%
- [ ] Other: ____________

Overall, was the app/intervention effective? *

- [ ] yes: all primary outcomes were significantly better in intervention group vs control
- [ ] partly: SOME primary outcomes were significantly better in intervention group vs control
- [x] no statistically significant difference between control and intervention
- [ ] potentially harmful: control was significantly better than intervention in one or more outcomes
- [ ] inconclusive: more research is needed
- [ ] Other: ____________

Article Preparation Status/Stage *

At which stage in your article preparation are you currently (at the time you fill in this form)

- ○ not submitted yet - in early draft status
- ○ not submitted yet - in late draft status, just before submission
- ○ submitted to a journal but not reviewed yet
- ◉ submitted to a journal and after receiving initial reviewer comments
- ○ submitted to a journal and accepted, but not published yet
- ○ published
- ○ Other:

Journal *

If you already know where you will submit this paper (or if it is already submitted), please provide the journal name (if it is not JMIR, provide the journal name under "other")

- ○ not submitted yet / unclear where I will submit this
- ○ Journal of Medical Internet Research (JMIR)
- ○ JMIR mHealth and UHealth
- ○ JMIR Serious Games
- ○ JMIR Mental Health
- ○ JMIR Public Health
- ○ JMIR Formative Research
- ○ Other JMIR sister journal
- ◉ Other: JMIR Medical Education

Is this a full powered effectiveness trial or a pilot/feasibility trial? *

- ( ) Pilot/feasibility
- (•) Fully powered

Manuscript tracking number *

If this is a JMIR submission, please provide the manuscript tracking number under "other" (The ms tracking number can be found in the submission acknowledgement email, or when you login as author in JMIR. If the paper is already published in JMIR, then the ms tracking number is the four-digit number at the end of the DOI, to be found at the bottom of each published article in JMIR)

- ( ) no ms number (yet) / not (yet) submitted to / published in JMIR
- (•) Other: 80097-1194452-5-SM

TITLE AND ABSTRACT

1a) TITLE: Identification as a randomized trial in the title

1a) Does your paper address CONSORT item 1a? *

I.e does the title contain the phrase "Randomized Controlled Trial"? (if not, explain the reason under "other")

- (•) yes
- ( ) Other:

1a-i) Identify the mode of delivery in the title

Identify the mode of delivery. Preferably use “web-based” and/or “mobile” and/or “electronic game” in the title. Avoid ambiguous terms like “online”, “virtual”, “interactive”. Use “Internet-based” only if Intervention includes non-web-based Internet components (e.g. email), use “computer-based” or “electronic” only if offline products are used. Use “virtual” only in the context of “virtual reality” (3-D worlds). Use “online” only in the context of “online support groups”. Complement or substitute product names with broader terms for the class of products (such as “mobile” or “smart phone” instead of “iphone”), especially if the application runs on different platforms.

	1	2	3	4	5	
subitem not at all important	○	○	○	○	◉	essential

Clear selection

Does your paper address subitem 1a-i? *

Copy and paste relevant sections from manuscript title (include quotes in quotation marks "like this" to indicate direct quotes from your manuscript), or elaborate on this item by providing additional information not in the ms, or briefly explain why the item is not applicable/relevant for your study

Web-Based Virtual Environment Versus Face-to-Face Delivery of Team-Based Learning for Anesthesia Techniques in Undergraduate Medical Students: A Randomized Controlled Trial

1a-ii) Non-web-based components or important co-interventions in title

Mention non-web-based components or important co-interventions in title, if any (e.g., “with telephone support”).

	1	2	3	4	5	
subitem not at all important	○	○	○	○	◉	essential

Clear selection

Does your paper address subitem 1a-ii?

Copy and paste relevant sections from manuscript title (include quotes in quotation marks "like this" to indicate direct quotes from your manuscript), or elaborate on this item by providing additional information not in the ms, or briefly explain why the item is not applicable/relevant for your study

Web-Based Virtual Environment Versus Face-to-Face Delivery of Team-Based Learning for Anesthesia Techniques in Undergraduate Medical Students: A Randomized Controlled Trial

1a-iii) Primary condition or target group in the title

Mention primary condition or target group in the title, if any (e.g., “for children with Type I Diabetes”) Example: A Web-based and Mobile Intervention with Telephone Support for Children with Type I Diabetes: Randomized Controlled Trial

	1	2	3	4	5	
subitem not at all important	○	○	○	○	◉	essential

Clear selection

Does your paper address subitem 1a-iii? *

Copy and paste relevant sections from manuscript title (include quotes in quotation marks "like this" to indicate direct quotes from your manuscript), or elaborate on this item by providing additional information not in the ms, or briefly explain why the item is not applicable/relevant for your study

Web-Based Virtual Environment Versus Face-to-Face Delivery of Team-Based Learning for Anesthesia Techniques in Undergraduate Medical Students: A Randomized Controlled Trial

1b) ABSTRACT: Structured summary of trial design, methods, results, and conclusions

NPT extension: Description of experimental treatment, comparator, care providers, centers, and blinding status.

1b-i) Key features/functionalities/components of the intervention and comparator in the METHODS section of the ABSTRACT

Mention key features/functionalities/components of the intervention and comparator in the abstract. If possible, also mention theories and principles used for designing the site. Keep in mind the needs of systematic reviewers and indexers by including important synonyms. (Note: Only report in the abstract what the main paper is reporting. If this information is missing from the main body of text, consider adding it)

	1	2	3	4	5	
subitem not at all important	○	○	○	○	◉	essential

Clear selection

Does your paper address subitem 1b-i? *

Copy and paste relevant sections from the manuscript abstract (include quotes in quotation marks "like this" to indicate direct quotes from your manuscript), or elaborate on this item by providing additional information not in the ms, or briefly explain why the item is not applicable/relevant for your study

We conducted a randomized, controlled, assessor-blinded trial at a Thai medical school (August 2024–January 2025). Fifth-year medical students who enrolled in an anesthesia techniques course were allocated to WBVE (Spatial®) or F2F sessions. Both groups received identical ten-module content in a standardized TBL sequence lasting 130 minutes. Individual knowledge was assessed before and after the session using a 15-item multiple-choice questionnaire via Google Forms. Satisfaction was measured immediately after class on a 5-point Likert scale.

1b-ii) Level of human involvement in the METHODS section of the ABSTRACT

Clarify the level of human involvement in the abstract, e.g., use phrases like “fully automated” vs. “therapist/nurse/care provider/physician-assisted” (mention number and expertise of providers involved, if any). (Note: Only report in the abstract what the main paper is reporting. If this information is missing from the main body of text, consider adding it)

	1	2	3	4	5	
subitem not at all important	○	○	○	○	◉	essential

Clear selection

Does your paper address subitem 1b-ii?

Copy and paste relevant sections from the manuscript abstract (include quotes in quotation marks "like this" to indicate direct quotes from your manuscript), or elaborate on this item by providing additional information not in the ms, or briefly explain why the item is not applicable/relevant for your study

We conducted a randomized, controlled, assessor-blinded trial at a Thai medical school (August 2024–January 2025). Fifth-year medical students who enrolled in an anesthesia techniques course were allocated to WBVE (Spatial®) or F2F sessions. Both groups received identical ten-module content in a standardized TBL sequence lasting 130 minutes. Individual knowledge was assessed before and after the session using a 15-item multiple-choice questionnaire via Google Forms. Satisfaction was measured immediately after class on a 5-point Likert scale.

1b-iii) Open vs. closed, web-based (self-assessment) vs. face-to-face assessments in the METHODS section of the ABSTRACT

Mention how participants were recruited (online vs. offline), e.g., from an open access website or from a clinic or a closed online user group (closed usergroup trial), and clarify if this was a purely web-based trial, or there were face-to-face components (as part of the intervention or for assessment). Clearly say if outcomes were self-assessed through questionnaires (as common in web-based trials). Note: In traditional offline trials, an open trial (open-label trial) is a type of clinical trial in which both the researchers and participants know which treatment is being administered. To avoid confusion, use “blinded” or “unblinded” to indicated the level of blinding instead of “open”, as “open” in web-based trials usually refers to “open access” (i.e. participants can self-enrol). (Note: Only report in the abstract what the main paper is reporting. If this information is missing from the main body of text, consider adding it)

	1	2	3	4	5	
subitem not at all important	○	○	○	○	◉	essential

Clear selection

Does your paper address subitem 1b-iii?

Copy and paste relevant sections from the manuscript abstract (include quotes in quotation marks "like this" to indicate direct quotes from your manuscript), or elaborate on this item by providing additional information not in the ms, or briefly explain why the item is not applicable/relevant for your study

We conducted a randomized, controlled, assessor-blinded trial at a Thai medical school (August 2024–January 2025). Fifth-year medical students who enrolled in an anesthesia techniques course were allocated to WBVE (Spatial®) or F2F sessions. Both groups received identical ten-module content in a standardized TBL sequence lasting 130 minutes. Individual knowledge was assessed before and after the session using a 15-item multiple-choice questionnaire via Google Forms. Satisfaction was measured immediately after class on a 5-point Likert scale.

1b-iv) RESULTS section in abstract must contain use data

Report number of participants enrolled/assessed in each group, the use/uptake of the intervention (e.g., attrition/adherence metrics, use over time, number of logins etc.), in addition to primary/secondary outcomes. (Note: Only report in the abstract what the main paper is reporting. If this information is missing from the main body of text, consider adding it)

	1	2	3	4	5	
subitem not at all important	○	○	○	○	◉	essential

Clear selection

Does your paper address subitem 1b-iv?

Copy and paste relevant sections from the manuscript abstract (include quotes in quotation marks "like this" to indicate direct quotes from your manuscript), or elaborate on this item by providing additional information not in the ms, or briefly explain why the item is not applicable/relevant for your study

Seventy-eight students were included in the study (F2F n=38; WBVE n=40). Post-class knowledge did not differ significantly (F2F: 11.24 (1.93); WBVE: 10.40 (2.62)). The mean difference was 0.88 (95% CI, -0.18 to 1.94) with P=.12. Learner satisfaction favored F2F across multiple domains, including overall course satisfaction. The mean difference was 0.42 (95% CI, 0.07 to 0.77), with P=.01. Both groups ran as planned. The WBVE group did not experience any technical problems.

1b-v) CONCLUSIONS/DISCUSSION in abstract for negative trials

Conclusions/Discussions in abstract for negative trials: Discuss the primary outcome - if the trial is negative (primary outcome not changed), and the intervention was not used, discuss whether negative results are attributable to lack of uptake and discuss reasons. (Note: Only report in the abstract what the main paper is reporting. If this information is missing from the main body of text, consider adding it)

	1	2	3	4	5	
subitem not at all important	○	○	○	○	◉	essential

Clear selection

Does your paper address subitem 1b-v?

Copy and paste relevant sections from the manuscript abstract (include quotes in quotation marks "like this" to indicate direct quotes from your manuscript), or elaborate on this item by providing additional information not in the ms, or briefly explain why the item is not applicable/relevant for your study

WBVE-delivered TBL achieved knowledge outcomes comparable to F2F delivery. However, participants were more satisfied with F2F classes. In settings constrained by limited physical space, large numbers of learners, or tight schedules, WBVEs may still be a practical alternative that preserves similar knowledge outcomes. Because students were more satisfied with F2F classes, WBVEs need some refinement rather than a simple replacement. Instructors must adapt their facilitation to the virtual setting, and technical barriers should be minimized.

INTRODUCTION

2a) In INTRODUCTION: Scientific background and explanation of rationale

2a-i) Problem and the type of system/solution

Describe the problem and the type of system/solution that is object of the study: intended as stand-alone intervention vs. incorporated in broader health care program? Intended for a particular patient population? Goals of the intervention, e.g., being more cost-effective to other interventions, replace or complement other solutions? (Note: Details about the intervention are provided in “Methods” under 5)

	1	2	3	4	5	
subitem not at all important	○	○	○	○	◉	essential

Clear selection

Does your paper address subitem 2a-i? *

Copy and paste relevant sections from the manuscript (include quotes in quotation marks "like this" to indicate direct quotes from your manuscript), or elaborate on this item by providing additional information not in the ms, or briefly explain why the item is not applicable/relevant for your study

However, comparative evidence remains limited. Existing studies are constrained by variability in reporting, small sample sizes, and non-randomized designs, particularly in anesthesia education [15-17]. Moreover, learners' diverse preferences with learning styles should be carefully considered when applying the WEVEs [18]. These limitations justify a randomized trial that isolates delivery mode by comparing a WBVE with face-to-face (F2F) instruction under an equivalent TBL design. We conducted a randomized controlled trial comparing a WBVE (Spatial® [19]) with F2F delivery of the same TBL curriculum in anesthesia techniques among fifth-year medical students. Both techniques used identical objectives, materials, facilitation, and assessments. Only the delivery mode differed. The primary outcome was post-class knowledge, and the secondary outcome was learner satisfaction with the learning process.

2a-ii) Scientific background, rationale: What is known about the (type of) system

Scientific background, rationale: What is known about the (type of) system that is the object of the study (be sure to discuss the use of similar systems for other conditions/diagnoses, if appropiate), motivation for the study, i.e. what are the reasons for and what is the context for this specific study, from which stakeholder viewpoint is the study performed, potential impact of findings [2]. Briefly justify the choice of the comparator.

	1	2	3	4	5	
subitem not at all important	○	○	○	○	◉	essential

Clear selection

Does your paper address subitem 2a-ii? *

Copy and paste relevant sections from the manuscript (include quotes in quotation marks "like this" to indicate direct quotes from your manuscript), or elaborate on this item by providing additional information not in the ms, or briefly explain why the item is not applicable/relevant for your study

Foundational knowledge of anesthesia techniques is core for medical students. Medical students are required to know about anesthesia administration, pharmacology, procedures, complication management, and interpretation of anesthesia records. Traditional didactic lectures offer a clear, efficient way to organize and deliver this content. It also allows broad coverage of key concepts in a limited time. However, limited interactivity can reduce engagement and encourage passive learning [1]. Interactive lectures address these limits with small-group discussion, problem-solving, and simulations. These activities create a more active classroom and support clinical reasoning. It also improves knowledge retention and provides immediate feedback to guide learning [2,3].

Team-based learning (TBL) is an effective approach to promote collaborative and active learning. TBL links to constructivist learning theory, the Interactive-Constructive-Active-Passive framework, and cognitive load theory. A simple sequence includes individual and team readiness assurance, application exercises, and immediate feedback. Students can move beyond just completing tasks to engaging with the content and peers. Additionally, it encourages a deeper understanding of concepts through discussion. Students can focus on what matters, think more deeply, and retain knowledge longer [4-6]. However, TBL also has its limitations. Challenges include managing group dynamics, resolving conflicts arising from differing opinions, managing time, and distributing workload. It also requires appropriate activity design, resources, and assessments [3,7,8].

Interactive lectures and TBL are used to facilitate the topic of anesthesia techniques for fifth-year medical students in our department. This learning technique demonstrates motivation, problem-based solving, and communication. Advances in medical education technology, such as web-based virtual environments (WBVEs), can support synchronous interaction, shared workspaces, and simulation-like experiences. The WBVEs are virtual worlds that enable real-time interactions between users and digital objects through technologies such as virtual and augmented reality. It provides immersive experiences, facilitates collaboration, and supports interactive and realistic simulations [9-13]. Compared with conventional TBL, WBVEs may make classes easier to join, easier to expand to more students, and more supportive of group work [7,12,14].

2b) In INTRODUCTION: Specific objectives or hypotheses

Does your paper address CONSORT subitem 2b? *

Copy and paste relevant sections from the manuscript (include quotes in quotation marks "like this" to indicate direct quotes from your manuscript), or elaborate on this item by providing additional information not in the ms, or briefly explain why the item is not applicable/relevant for your study

However, comparative evidence remains limited. Existing studies are constrained by variability in reporting, small sample sizes, and non-randomized designs, particularly in anesthesia education [15-17]. Moreover, learners' diverse preferences with learning styles should be carefully considered when applying the WEVEs [18]. These limitations justify a randomized trial that isolates delivery mode by comparing a WBVE with face-to-face (F2F) instruction under an equivalent TBL design. We conducted a randomized controlled trial comparing a WBVE (Spatial® [19]) with F2F delivery of the same TBL curriculum in anesthesia techniques among fifth-year medical students. Both techniques used identical objectives, materials, facilitation, and assessments. Only the delivery mode differed. The primary outcome was post-class knowledge, and the secondary outcome was learner satisfaction with the learning process.

METHODS

3a) Description of trial design (such as parallel, factorial) including allocation ratio

Does your paper address CONSORT subitem 3a? *

Copy and paste relevant sections from the manuscript (include quotes in quotation marks "like this" to indicate direct quotes from your manuscript), or elaborate on this item by providing additional information not in the ms, or briefly explain why the item is not applicable/relevant for your study

This study was a randomized, controlled, single-blinded trial. Double blinding was not feasible, as participants were aware of their assigned groups.

3b) Important changes to methods after trial commencement (such as eligibility criteria), with reasons

Does your paper address CONSORT subitem 3b? *

Copy and paste relevant sections from the manuscript (include quotes in quotation marks "like this" to indicate direct quotes from your manuscript), or elaborate on this item by providing additional information not in the ms, or briefly explain why the item is not applicable/relevant for your study

No important changes to the trial methods, eligibility criteria, or outcomes were made after trial commencement.

3b-i) Bug fixes, Downtimes, Content Changes

Bug fixes, Downtimes, Content Changes: ehealth systems are often dynamic systems. A description of changes to methods therefore also includes important changes made on the intervention or comparator during the trial (e.g., major bug fixes or changes in the functionality or content) (5-iii) and other “unexpected events” that may have influenced study design such as staff changes, system failures/downtimes, etc. [2].

	1	2	3	4	5	
subitem not at all important	○	○	○	○	◉	essential

Clear selection

Does your paper address subitem 3b-i?

Copy and paste relevant sections from the manuscript (include quotes in quotation marks "like this" to indicate direct quotes from your manuscript), or elaborate on this item by providing additional information not in the ms, or briefly explain why the item is not applicable/relevant for your study

Both groups ran as planned. The WBVE group did not experience any technical problems.

4a) Eligibility criteria for participants

Does your paper address CONSORT subitem 4a? *

Copy and paste relevant sections from the manuscript (include quotes in quotation marks "like this" to indicate direct quotes from your manuscript), or elaborate on this item by providing additional information not in the ms, or briefly explain why the item is not applicable/relevant for your study

We recruited fifth-year medical students from the Faculty of Medicine, Khon Kaen University, who attended the Anesthesiology course at the Department of Anesthesiology. Students who had previously completed the Anesthesiology course or were unable to comply with the study protocol were excluded.

4a-i) Computer / Internet literacy

Computer / Internet literacy is often an implicit "de facto" eligibility criterion - this should be explicitly clarified.

	1	2	3	4	5	
subitem not at all important	○	○	○	○	◉	essential

Clear selection

Does your paper address subitem 4a-i?

Copy and paste relevant sections from the manuscript (include quotes in quotation marks "like this" to indicate direct quotes from your manuscript), or elaborate on this item by providing additional information not in the ms, or briefly explain why the item is not applicable/relevant for your study

There was no formal computer literacy test. All students had sufficient experience to use standard university computers and Google Classroom. Participants were regular fifth-year medical students using the university's IT infrastructure.

4a-ii) Open vs. closed, web-based vs. face-to-face assessments:

Open vs. closed, web-based vs. face-to-face assessments: Mention how participants were recruited (online vs. offline), e.g., from an open access website or from a clinic, and clarify if this was a purely web-based trial, or there were face-to-face components (as part of the intervention or for assessment), i.e., to what degree got the study team to know the participant. In online-only trials, clarify if participants were quasi-anonymous and whether having multiple identities was possible or whether technical or logistical measures (e.g., cookies, email confirmation, phone calls) were used to detect/prevent these.

	1	2	3	4	5	
subitem not at all important	○	○	○	○	◉	essential

Clear selection

Does your paper address subitem 4a-ii? *

Copy and paste relevant sections from the manuscript (include quotes in quotation marks "like this" to indicate direct quotes from your manuscript), or elaborate on this item by providing additional information not in the ms, or briefly explain why the item is not applicable/relevant for your study

Sessions ran on the Spatial® platform (Spatial Systems, Inc., USA; Thai localization) [19] using desktop computers on the university's secure local network. On first login, students created an account and entered a virtual space with three areas (a classroom, an operating room, and a common room).

4a-iii) Information giving during recruitment

Information given during recruitment. Specify how participants were briefed for recruitment and in the informed consent procedures (e.g., publish the informed consent documentation as appendix, see also item X26), as this information may have an effect on user self-selection, user expectation and may also bias results.

	1	2	3	4	5	
subitem not at all important	○	○	○	○	◉	essential

Clear selection

Does your paper address subitem 4a-iii?

Copy and paste relevant sections from the manuscript (include quotes in quotation marks "like this" to indicate direct quotes from your manuscript), or elaborate on this item by providing additional information not in the ms, or briefly explain why the item is not applicable/relevant for your study

We used cluster randomization at the class level to minimize contamination between teaching groups. Students were grouped into clusters based on their scheduled learning sessions. Each cluster comprised approximately 20 students. Clusters were then randomly allocated in a 1:1 ratio to either the WBVE or F2F group using computer-generated random numbers. We used a block size of 2 to ensure that each group had a similar number of clusters. The randomization sequence was generated and implemented by investigators who were not involved in teaching or assessment to reduce the risk of allocation bias. For clusters randomized to the WBVE group, students who decline research participation will receive F2F teaching delivered by another instructor, scheduled at the same time and following an identical teaching plan. For clusters randomized to the F2F group, students who decline research participation will still attend the same F2F session with the same instructor but will not be asked to complete the post-test or satisfaction questionnaire.

4b) Settings and locations where the data were collected

Does your paper address CONSORT subitem 4b? *

Copy and paste relevant sections from the manuscript (include quotes in quotation marks "like this" to indicate direct quotes from your manuscript), or elaborate on this item by providing additional information not in the ms, or briefly explain why the item is not applicable/relevant for your study

We recruited fifth-year medical students from the Faculty of Medicine, Khon Kaen University, who attended the Anesthesiology course at the Department of Anesthesiology. Students who had previously completed the Anesthesiology course or were unable to comply with the study protocol were excluded. All participants provided written informed consent. The study was conducted from August 26, 2024, to January 7, 2025. Withdrawal criteria included technical issues, such as internet disruptions or software glitches, on the Spatial® [19].

4b-i) Report if outcomes were (self-)assessed through online questionnaires

Clearly report if outcomes were (self-)assessed through online questionnaires (as common in web-based trials) or otherwise.

	1	2	3	4	5	
subitem not at all important	○	○	○	○	●	essential

Clear selection

Does your paper address subitem 4b-i? *

Copy and paste relevant sections from the manuscript (include quotes in quotation marks "like this" to indicate direct quotes from your manuscript), or elaborate on this item by providing additional information not in the ms, or briefly explain why the item is not applicable/relevant for your study

Immediately after the teaching session, 15 minutes have been arranged for outcome measurement. All students completed a 15 MCQ posttest via Google Forms, using the exact test specification as the pretest, under invigilated conditions. They then completed a satisfaction questionnaire in Google Forms (five-point Likert scale; instrument and scoring details are provided in the Supplement). The forms were identical for both groups and were accessed through locked links. Answer keys were withheld until all submissions had been received. In addition, we enabled automatic scoring and timestamp logging, restricted responses to a single submission per account, and exported raw data directly from Google Forms into the analysis dataset.

4b-ii) Report how institutional affiliations are displayed

Report how institutional affiliations are displayed to potential participants [on ehealth media], as affiliations with prestigious hospitals or universities may affect volunteer rates, use, and reactions with regards to an intervention.(Not a required item – describe only if this may bias results)

	1	2	3	4	5	
subitem not at all important	○	○	○	○	●	essential

Clear selection

Does your paper address subitem 4b-ii?

Copy and paste relevant sections from the manuscript (include quotes in quotation marks "like this" to indicate direct quotes from your manuscript), or elaborate on this item by providing additional information not in the ms, or briefly explain why the item is not applicable/relevant for your study

We recruited fifth-year medical students from the Faculty of Medicine, Khon Kaen University, who attended the Anesthesiology course at the Department of Anesthesiology.

5) The interventions for each group with sufficient details to allow replication, including how and when they were actually administered

5-i) Mention names, credential, affiliations of the developers, sponsors, and owners

Mention names, credential, affiliations of the developers, sponsors, and owners [6] (if authors/evaluators are owners or developer of the software, this needs to be declared in a "Conflict of interest" section or mentioned elsewhere in the manuscript).

	1	2	3	4	5	
subitem not at all important	○	○	○	○	◉	essential

Clear selection

Does your paper address subitem 5-i?

Copy and paste relevant sections from the manuscript (include quotes in quotation marks "like this" to indicate direct quotes from your manuscript), or elaborate on this item by providing additional information not in the ms, or briefly explain why the item is not applicable/relevant for your study

from the Faculty of Medicine, Khon Kaen University, who attended the Anesthesiology course at the Department of Anesthesiology.

5-ii) Describe the history/development process

Describe the history/development process of the application and previous formative evaluations (e.g., focus groups, usability testing), as these will have an impact on adoption/use rates and help with interpreting results.

	1	2	3	4	5	
subitem not at all important	○	○	○	○	◉	essential

Clear selection

Does your paper address subitem 5-ii?

Copy and paste relevant sections from the manuscript (include quotes in quotation marks "like this" to indicate direct quotes from your manuscript), or elaborate on this item by providing additional information not in the ms, or briefly explain why the item is not applicable/relevant for your study

Supplement
3.2 WBVE delivery TBL lesson plan
This Spatial composed of two rooms. One is a basic concept room, and the other is an operations room (with four small rooms inside).
Each team must complete the questions to achieve the case-solving aim for each learning content.
The only route to encounter the content of each topic sequentially.
Each person will have a login and either a laptop or a tablet. It is advisable to have headphones.
Travel together in groups, but with four separate routes and identical content for each group.
Assistance is available through the ability to ask the teacher questions when needed.
The content is divided into ten segments, and there will be question-answering sessions where each person has their answer but works together. Aggregate group scores from the questions answered.
Content 1: Introduction to anesthesia technique 2 minutes (VDO-narrative presentation) + Response to surgical stimuli 2 minutes (cartoon) (VDO-narrative presentation) + Anesthesia definition and benefit of anesthesia 2 minutes (VDO-narrative presentation)
Content 2: Level of anesthesia and standard care 15 minutes (VDO-narrative presentation, 8 MCQs)
Content 3: Anesthetic agent (intravenous anesthetic drugs): mechanism, action, dose, benefits, and risks 15 minutes (VDO-narrative presentation, 2 MCQs)
Content 4: Anesthetic agent (inhalation anesthetic drugs): mechanism, action, dose, benefits, and risks 15 minutes (VDO-narrative presentation, 2 MCQs)
Content 5: Anesthetic agent (muscle relaxants): mechanism, action, dose, benefits, and risks 15 minutes (VDO-narrative presentation, 2 MCQs)
Content 6: Anesthesia technique (concept and type) 15 minutes (VDO-narrative presentation, 4 MCQs)
Content 7: Complication 5 minutes (VDO-narrative presentation)
Content 8: Anesthesia records 9 minutes (VDO-narrative presentation)
Content 9 (4 case studies, I, J, K, and L): How to interpret the anesthetic record, go to four operating rooms, see the anesthetic record, answer the questions, 30 minutes (12 MCQs)
Content 10: Take-home message, conclusion for every topic, 5 minutes
During the final 15 minutes of the class
The instructor summarizes all of the content covered by asking the medical students to summarize according to the following points jointly:
Explain the principles and benefits of anesthesia.
Describe the different levels of anesthesia/sedation (minimal sedation, moderate sedation, deep sedation, general anesthesia).
Explain the pharmacology of drugs used for anesthesia, including intravenous agents, inhalation agents, and muscle relaxants.
Classify the clinical techniques for administering anesthesia and describe the perioperative patient management (TIVA, balanced general anesthesia, inhalational general anesthesia, VIMA).
Identify complications arising from the administration of anesthetic drugs.

5-iii) Revisions and updating

Revisions and updating. Clearly mention the date and/or version number of the application/intervention (and comparator, if applicable) evaluated, or describe whether the intervention underwent major changes during the evaluation process, or whether the development and/or content was “frozen” during the trial. Describe dynamic components such as news feeds or changing content which may have an impact on the replicability of the intervention (for unexpected events see item 3b).

	1	2	3	4	5	
subitem not at all important	○	○	○	○	◉	essential

Clear selection

Does your paper address subitem 5-iii?

Copy and paste relevant sections from the manuscript (include quotes in quotation marks "like this" to indicate direct quotes from your manuscript), or elaborate on this item by providing additional information not in the ms, or briefly explain why the item is not applicable/relevant for your study

Supplement
No major revisions to the WBVE occurred during August 2024–January 2025; the system and materials were frozen for the trial.
WBVE group did not experience any technical problems.

5-iv) Quality assurance methods

Provide information on quality assurance methods to ensure accuracy and quality of information provided [1], if applicable.

	1	2	3	4	5	
subitem not at all important	○	○	○	○	◉	essential

Clear selection

Does your paper address subitem 5-iv?

Copy and paste relevant sections from the manuscript (include quotes in quotation marks "like this" to indicate direct quotes from your manuscript), or elaborate on this item by providing additional information not in the ms, or briefly explain why the item is not applicable/relevant for your study

Supplement
Teaching content was aligned with the existing anesthesia curriculum and reviewed by the anesthesiology faculty. Quality was ensured mainly via content and instrument validation. The test specification table is provided in the Supplement.

5-v) Ensure replicability by publishing the source code, and/or providing screenshots/screen-capture video, and/or providing flowcharts of the algorithms used

Ensure replicability by publishing the source code, and/or providing screenshots/screen-capture video, and/or providing flowcharts of the algorithms used. Replicability (i.e., other researchers should in principle be able to replicate the study) is a hallmark of scientific reporting.

	1	2	3	4	5	
subitem not at all important	○	○	○	○	◉	essential

Clear selection

Does your paper address subitem 5-v?

Copy and paste relevant sections from the manuscript (include quotes in quotation marks "like this" to indicate direct quotes from your manuscript), or elaborate on this item by providing additional information not in the ms, or briefly explain why the item is not applicable/relevant for your study

3.2 WBVE delivery TBL lesson plan in supplement

5-vi) Digital preservation

Digital preservation: Provide the URL of the application, but as the intervention is likely to change or disappear over the course of the years; also make sure the intervention is archived (Internet Archive, webcitation.org, and/or publishing the source code or screenshots/videos alongside the article). As pages behind login screens cannot be archived, consider creating demo pages which are accessible without login.

	1	2	3	4	5	
subitem not at all important	○	○	○	○	◉	essential

Clear selection

Does your paper address subitem 5-vi?

Copy and paste relevant sections from the manuscript (include quotes in quotation marks "like this" to indicate direct quotes from your manuscript), or elaborate on this item by providing additional information not in the ms, or briefly explain why the item is not applicable/relevant for your study

The intervention used the commercial Spatial® platform (Spatial Systems, Inc.; URL: https://www.spatial.io). Screenshots and a schematic of the virtual environment are included in Figure 1 and will remain as a permanent record in the published article and supplementary materials.

5-vii) Access

Access: Describe how participants accessed the application, in what setting/context, if they had to pay (or were paid) or not, whether they had to be a member of specific group. If known, describe how participants obtained “access to the platform and Internet” [1]. To ensure access for editors/reviewers/readers, consider to provide a “backdoor” login account or demo mode for reviewers/readers to explore the application (also important for archiving purposes, see vi).

	1	2	3	4	5	
subitem not at all important	○	○	○	○	◉	essential

Clear selection

Does your paper address subitem 5-vii? *

Copy and paste relevant sections from the manuscript (include quotes in quotation marks "like this" to indicate direct quotes from your manuscript), or elaborate on this item by providing additional information not in the ms, or briefly explain why the item is not applicable/relevant for your study

Participants accessed the intervention only during scheduled course sessions as enrolled students. They did not pay. There were no external users. Sessions ran on the Spatial® platform using desktop computers on the university's secure local network.

5-viii) Mode of delivery, features/functionalities/components of the intervention and comparator, and the theoretical framework

Describe mode of delivery, features/functionalities/components of the intervention and comparator, and the theoretical framework [6] used to design them (instructional strategy [1], behaviour change techniques, persuasive features, etc., see e.g., [7, 8] for terminology). This includes an in-depth description of the content (including where it is coming from and who developed it) [1]," whether [and how] it is tailored to individual circumstances and allows users to track their progress and receive feedback" [6]. This also includes a description of communication delivery channels and – if computer-mediated communication is a component – whether communication was synchronous or asynchronous [6]. It also includes information on presentation strategies [1], including page design principles, average amount of text on pages, presence of hyperlinks to other resources, etc. [1].

	1	2	3	4	5	
subitem not at all important	○	○	○	○	◉	essential

Clear selection

Does your paper address subitem 5-viii? *

Copy and paste relevant sections from the manuscript (include quotes in quotation marks "like this" to indicate direct quotes from your manuscript), or elaborate on this item by providing additional information not in the ms, or briefly explain why the item is not applicable/relevant for your study

Interventions
Pre-intervention process
After recruitment into the study, all participants in both groups were given access to the

learning material via online learning in Google Classroom (Google Inc., USA), including a slide presentation and supplementary video material, two days before the teaching session. On the teaching day, students first received a 5-minute briefing about study procedures and learning objectives. They then completed a 15-question multiple-choice (MCQ) pretest in 15 minutes to assess baseline knowledge. Participants also completed a brief questionnaire capturing student characteristics. The test specification table is provided in the Supplement.

Session allocation and team setup

After baseline assessments, students attended their assigned session (either a WBVE or an F2F session). In each group, students were organized into four team-based learning teams of 5–6 students, supervised by one instructor. Each session lasted 130 minutes.

Content and structure common to both groups

Both groups received identical content organized into ten modules on anesthesia techniques (listed in the Supplement). Each module began with core knowledge followed by a problem-solving scenario aligned with the same learning objectives. Students progressed sequentially through the modules to complete the required tasks.

Face-to-face group

The ten modules described above were delivered F2F using a standard TBL sequence. At the start of the session, the instructor reviewed the learning objectives and ground rules and confirmed team assignments. For each module (see Supplement), students first received a brief mini-lecture to consolidate core concepts. Subsequently, teams completed a scenario-based application task at their respective tables. They recorded a single team answer. They received immediate feedback, followed by a short whole-class debrief that compared rationales across teams. The room layout comprised four separated team tables with a central screen, and teams were asked to discuss quietly to minimize cross-talk. Materials were identical to those in the other group (handouts mirroring the slides and identical question stems). Any clarifying questions were addressed at the table before the debrief.

Web-based virtual environment group

Sessions ran on the Spatial® platform (Spatial Systems, Inc., USA; Thai localization) [19] using desktop computers on the university's secure local network. On first login, students created an account and entered a virtual space with three areas (a classroom, an operating room, and a common room). Each was equipped with detailed 3D anesthesia equipment to support the team's application tasks. The same ten interactive modules described above were delivered in sequence. Each module began with brief core content, followed by a scenario-based application task aligned with the learning objectives. Students moved through the environment with keyboard-controlled avatars and collaborated in real time via built-in voice and text chat. Embedded multimedia (short videos, slide decks, interactive cases, and simple educational games) supported engagement. The instructor monitored team rooms, offered real-time guidance, provided immediate feedback, and answered questions as they arose. Any clarifying questions were addressed in the common room before the debrief. WBVEs' detailed figure is provided in Figure 1.

Post-intervention process

Immediately after the teaching session, 15 minutes have been arranged for outcome measurement. All students completed a 15 MCQ posttest via Google Forms, using the exact test specification as the pretest, under invigilated conditions. They then completed a satisfaction questionnaire in Google Forms (five-point Likert scale; instrument and scoring details are provided in the Supplement). The forms were identical for both groups and were accessed through locked links. Answer keys were withheld until all submissions had been received. In addition, we enabled automatic scoring and timestamp logging, restricted

responses to a single submission per account, and exported raw data directly from Google Forms into the analysis dataset.

5-ix) Describe use parameters

Describe use parameters (e.g., intended "doses" and optimal timing for use). Clarify what instructions or recommendations were given to the user, e.g., regarding timing, frequency, heaviness of use, if any, or was the intervention used ad libitum.

	1	2	3	4	5	
subitem not at all important	○	○	○	○	◉	essential

Clear selection

Does your paper address subitem 5-ix?

Copy and paste relevant sections from the manuscript (include quotes in quotation marks "like this" to indicate direct quotes from your manuscript), or elaborate on this item by providing additional information not in the ms, or briefly explain why the item is not applicable/relevant for your study

Each session lasted 130 minutes. The recommended dose was one 130-minute TBL session plus pre-class online materials.
After recruitment, participants were given access to the learning materials via online learning in Google Classroom 2 days before the teaching session.

5-x) Clarify the level of human involvement

Clarify the level of human involvement (care providers or health professionals, also technical assistance) in the e-intervention or as co-intervention (detail number and expertise of professionals involved, if any, as well as "type of assistance offered, the timing and frequency of the support, how it is initiated, and the medium by which the assistance is delivered". It may be necessary to distinguish between the level of human involvement required for the trial, and the level of human involvement required for a routine application outside of a RCT setting (discuss under item 21 – generalizability).

	1	2	3	4	5	
subitem not at all important	○	○	○	○	◉	essential

Clear selection

Does your paper address subitem 5-x?

Copy and paste relevant sections from the manuscript (include quotes in quotation marks "like this" to indicate direct quotes from your manuscript), or elaborate on this item by providing additional information not in the ms, or briefly explain why the item is not applicable/relevant for your study

After baseline assessments, students attended their assigned session (either a WBVE or an F2F session). In each group, students were organized into four team-based learning teams of 5–6 students, supervised by one instructor. Each session lasted 130 minutes.

5-xi) Report any prompts/reminders used

Report any prompts/reminders used: Clarify if there were prompts (letters, emails, phone calls, SMS) to use the application, what triggered them, frequency etc. It may be necessary to distinguish between the level of prompts/reminders required for the trial, and the level of prompts/reminders for a routine application outside of a RCT setting (discuss under item 21 – generalizability).

	1	2	3	4	5	
subitem not at all important	○	○	○	○	◉	essential

Clear selection

Does your paper address subitem 5-xi? *

Copy and paste relevant sections from the manuscript (include quotes in quotation marks "like this" to indicate direct quotes from your manuscript), or elaborate on this item by providing additional information not in the ms, or briefly explain why the item is not applicable/relevant for your study

No email/SMS prompts to use the app were used; students attended a scheduled, invigilated teaching session. There were no automated digital reminders. Participation was organized as a compulsory course session:
The study was conducted from August 26, 2024, to January 7, 2025, during the scheduled anesthesiology course.

5-xii) Describe any co-interventions (incl. training/support)

Describe any co-interventions (incl. training/support): Clearly state any interventions that are provided in addition to the targeted eHealth intervention, as ehealth intervention may not be designed as stand-alone intervention. This includes training sessions and support [1]. It may be necessary to distinguish between the level of training required for the trial, and the level of training for a routine application outside of a RCT setting (discuss under item 21 – generalizability.

	1	2	3	4	5	
subitem not at all important	○	○	○	○	◉	essential

Clear selection

Does your paper address subitem 5-xii? *

Copy and paste relevant sections from the manuscript (include quotes in quotation marks "like this" to indicate direct quotes from your manuscript), or elaborate on this item by providing additional information not in the ms, or briefly explain why the item is not applicable/relevant for your study

Both groups received identical content organized into ten modules on anesthesia techniques (listed in the Supplement). Each module began with core knowledge followed by a problem-solving scenario aligned with the same learning objectives. Students progressed sequentially through the modules to complete the required tasks.

6a) Completely defined pre-specified primary and secondary outcome measures, including how and when they were assessed

Does your paper address CONSORT subitem 6a? *

Copy and paste relevant sections from the manuscript (include quotes in quotation marks "like this" to indicate direct quotes from your manuscript), or elaborate on this item by providing additional information not in the ms, or briefly explain why the item is not applicable/relevant for your study

A 15-item MCQ pre-/posttest measured knowledge, and a 21-item Likert-scale questionnaire measured satisfaction.
The primary outcome was post-class knowledge, and the secondary outcome was learner satisfaction with the learning process.

6a-i) Online questionnaires: describe if they were validated for online use and apply CHERRIES items to describe how the questionnaires were designed/deployed

If outcomes were obtained through online questionnaires, describe if they were validated for online use and apply CHERRIES items to describe how the questionnaires were designed/deployed [9].

	1	2	3	4	5	
subitem not at all important	○	○	○	○	◉	essential

Clear selection

Does your paper address subitem 6a-i?

Copy and paste relevant sections from manuscript text

Pre- and post-intervention knowledge evaluations were assessed using a 15-item MCQ test mapped to a test specification table (see Supplement). The authors designed and developed a questionnaire on students' satisfaction with the learning process, drawing on previous studies [21,22], and adjusted its content for use in a Thai context (see Supplement). Student satisfaction was evaluated immediately after the intervention using a 21-item survey, with responses on a 5-point Likert scale (1=strongly disagree, 2=disagree, 3=neither agree nor disagree, 4=agree, 5=strongly agree). The questionnaire's content validity was confirmed by three experts. Internal consistency was evaluated using Cronbach's alpha, with $\alpha = 0.95$. We also recorded gender, age, Grade Point Average (GPA), and experience with WBVEs (including frequency, proficiency, and comfort).

6a-ii) Describe whether and how "use" (including intensity of use/dosage) was defined/measured/monitored

Describe whether and how "use" (including intensity of use/dosage) was defined/measured/monitored (logins, logfile analysis, etc.). Use/adoption metrics are important process outcomes that should be reported in any ehealth trial.

	1	2	3	4	5	
subitem not at all important	○	○	○	○	◉	essential

Clear selection

Does your paper address subitem 6a-ii?

Copy and paste relevant sections from manuscript text

Because the intervention consisted of a single, scheduled TBL session delivered in a classroom/computer lab, use was defined as attending the session and completing the pre- and post-class questionnaires. All analyzed participants did so; detailed app log-file metrics were not collected.

6a-iii) Describe whether, how, and when qualitative feedback from participants was obtained

Describe whether, how, and when qualitative feedback from participants was obtained (e.g., through emails, feedback forms, interviews, focus groups).

	1	2	3	4	5	
subitem not at all important	○	○	○	○	◉	essential

Clear selection

Does your paper address subitem 6a-iii?

Copy and paste relevant sections from manuscript text

We did not collect formal qualitative feedback; only quantitative satisfaction ratings were obtained.

6b) Any changes to trial outcomes after the trial commenced, with reasons

Does your paper address CONSORT subitem 6b? *

Copy and paste relevant sections from the manuscript (include quotes in quotation marks "like this" to indicate direct quotes from your manuscript), or elaborate on this item by providing additional information not in the ms, or briefly explain why the item is not applicable/relevant for your study

The primary and secondary outcomes (post-class knowledge and learner satisfaction) were prespecified and not changed after trial commencement.

7a) How sample size was determined

NPT: When applicable, details of whether and how the clustering by care provides or centers was addressed

7a-i) Describe whether and how expected attrition was taken into account when calculating the sample size

Describe whether and how expected attrition was taken into account when calculating the sample size.

	1	2	3	4	5	
subitem not at all important	○	○	○	○	◉	essential

Clear selection

Does your paper address subitem 7a-i?

Copy and paste relevant sections from manuscript title (include quotes in quotation marks "like this" to indicate direct quotes from your manuscript), or elaborate on this item by providing additional information not in the ms, or briefly explain why the item is not applicable/relevant for your study

The required sample size was calculated based on a post-learning knowledge score of 57.94% with an SD of 14.07% in 63 fifth-year medical students who had taken anesthesiology for this topic in the previous academic year (2023). To detect a 10% difference in knowledge score (type I error of 0.05) with 80% power, we determined that a sample size of 32 participants in each group would be required. The dropout rate was accounted for in the sample size, which included at least 40 participants per group, representing approximately 10% of the total dropout rate.

7b) When applicable, explanation of any interim analyses and stopping guidelines

Does your paper address CONSORT subitem 7b? *

Copy and paste relevant sections from the manuscript (include quotes in quotation marks "like this" to indicate direct quotes from your manuscript), or elaborate on this item by providing additional information not in the ms, or briefly explain why the item is not applicable/relevant for your study

No interim analyses or stopping guidelines were specified; the trial ran as planned until all scheduled clusters had completed the intervention.

8a) Method used to generate the random allocation sequence
NPT: When applicable, how care providers were allocated to each trial group

Does your paper address CONSORT subitem 8a? *

Copy and paste relevant sections from the manuscript (include quotes in quotation marks "like this" to indicate direct quotes from your manuscript), or elaborate on this item by providing additional information not in the ms, or briefly explain why the item is not applicable/relevant for your study

Clusters were then randomly allocated in a 1:1 ratio to either the WBVE or F2F group using computer-generated random numbers.

8b) Type of randomisation; details of any restriction (such as blocking and block size)

Does your paper address CONSORT subitem 8b? *

Copy and paste relevant sections from the manuscript (include quotes in quotation marks "like this" to indicate direct quotes from your manuscript), or elaborate on this item by providing additional information not in the ms, or briefly explain why the item is not applicable/relevant for your study

We used cluster randomization at the class level. We used a block size of 2 to ensure that each group had a similar number of clusters.

9) Mechanism used to implement the random allocation sequence (such as sequentially numbered containers), describing any steps taken to conceal the sequence until interventions were assigned

Does your paper address CONSORT subitem 9? *

Copy and paste relevant sections from the manuscript (include quotes in quotation marks "like this" to indicate direct quotes from your manuscript), or elaborate on this item by providing additional information not in the ms, or briefly explain why the item is not applicable/relevant for your study

The randomization sequence was generated and implemented by investigators who were not involved in teaching or assessment to reduce the risk of allocation bias.

10) Who generated the random allocation sequence, who enrolled participants, and who assigned participants to interventions

Does your paper address CONSORT subitem 10? *

Copy and paste relevant sections from the manuscript (include quotes in quotation marks "like this" to indicate direct quotes from your manuscript), or elaborate on this item by providing additional information not in the ms, or briefly explain why the item is not applicable/relevant for your study

The randomization sequence was generated and implemented by investigators who were not involved in teaching or assessment to reduce the risk of allocation bias.

11a) If done, who was blinded after assignment to interventions (for example, participants, care providers, those assessing outcomes) and how

NPT: Whether or not administering co-interventions were blinded to group assignment

11a-i) Specify who was blinded, and who wasn’t

Specify who was blinded, and who wasn’t. Usually, in web-based trials it is not possible to blind the participants [1, 3] (this should be clearly acknowledged), but it may be possible to blind outcome assessors, those doing data analysis or those administering co-interventions (if any).

	1	2	3	4	5	
subitem not at all important	○	○	○	○	◉	essential

Clear selection

Does your paper address subitem 11a-i? *

Copy and paste relevant sections from the manuscript (include quotes in quotation marks "like this" to indicate direct quotes from your manuscript), or elaborate on this item by providing additional information not in the ms, or briefly explain why the item is not applicable/relevant for your study

Single-blinding refers to the blinding of outcome assessors/analysts; participants and instructors were not blinded.
This study was a randomized, controlled, single-blinded trial. Double blinding was not feasible, as participants were aware of their assigned groups.

11a-ii) Discuss e.g., whether participants knew which intervention was the “intervention of interest” and which one was the “comparator”

Informed consent procedures (4a-ii) can create biases and certain expectations - discuss e.g., whether participants knew which intervention was the “intervention of interest” and which one was the “comparator”.

	1	2	3	4	5	
subitem not at all important	○	○	○	○	◉	essential

Clear selection

Does your paper address subitem 11a-ii?

Copy and paste relevant sections from the manuscript (include quotes in quotation marks "like this" to indicate direct quotes from your manuscript), or elaborate on this item by providing additional information not in the ms, or briefly explain why the item is not applicable/relevant for your study

Students knew whether they were in WBVE or F2F. Both were presented as alternative TBL delivery modes within the standard course, without emphasizing one as “superior”.

11b) If relevant, description of the similarity of interventions

(this item is usually not relevant for ehealth trials as it refers to similarity of a placebo or sham intervention to a active medication/intervention)

Does your paper address CONSORT subitem 11b? *

Copy and paste relevant sections from the manuscript (include quotes in quotation marks "like this" to indicate direct quotes from your manuscript), or elaborate on this item by providing additional information not in the ms, or briefly explain why the item is not applicable/relevant for your study

Only the delivery mode differed. Both groups received identical content organized into ten modules on anesthesia techniques… Each module began with core knowledge followed by a problem-solving scenario aligned with the same learning objectives.

12a) Statistical methods used to compare groups for primary and secondary outcomes

NPT: When applicable, details of whether and how the clustering by care providers or centers was addressed

Does your paper address CONSORT subitem 12a? *

Copy and paste relevant sections from the manuscript (include quotes in quotation marks "like this" to indicate direct quotes from your manuscript), or elaborate on this item by providing additional information not in the ms, or briefly explain why the item is not applicable/relevant for your study

Data was analyzed using STATA/SE 18.0 for Windows (StataCorp LP, College Station, TX, USA). Descriptive statistics summarized participant characteristics. Categorical variables are presented as counts and percentages, and continuous variables are presented as means and standard deviations (SDs). Proportions were calculated using non-missing denominators. Analyses followed the intention-to-treat principle. Between-group comparisons were conducted using linear mixed modeling. We reported the mean differences and 95% confidence intervals (CIs). For the primary outcome (knowledge), we used posttest score as the dependent variable, study group as the factor, and pretest score as a covariate. For multiple tests that were analyzed, the false discovery rate was controlled at 5% (Benjamini–Hochberg method). Item-level Likert responses were summarized descriptively as means (SDs).

12a-i) Imputation techniques to deal with attrition / missing values

Imputation techniques to deal with attrition / missing values: Not all participants will use the intervention/comparator as intended and attrition is typically high in ehealth trials. Specify how participants who did not use the application or dropped out from the trial were treated in the statistical analysis (a complete case analysis is strongly discouraged, and simple imputation techniques such as LOCF may also be problematic [4]).

	1	2	3	4	5	
subitem not at all important	○	○	○	○	◉	essential

Clear selection

Does your paper address subitem 12a-i? *

Copy and paste relevant sections from the manuscript (include quotes in quotation marks "like this" to indicate direct quotes from your manuscript), or elaborate on this item by providing additional information not in the ms, or briefly explain why the item is not applicable/relevant for your study

Given only one missing data case, a complete-case analysis was appropriate. Seventy-nine participants were enrolled… One participant from the WBVE group was excluded due to incomplete data. Seventy-eight participants had complete data in the analysis (Figure 2).

12b) Methods for additional analyses, such as subgroup analyses and adjusted analyses

Does your paper address CONSORT subitem 12b? *

Copy and paste relevant sections from the manuscript (include quotes in quotation marks "like this" to indicate direct quotes from your manuscript), or elaborate on this item by providing additional information not in the ms, or briefly explain why the item is not applicable/relevant for your study

he same linear mixed model, with covariate adjustment and FDR control, was used for primary and secondary outcomes; no unplanned subgroup analyses were reported.

X26) REB/IRB Approval and Ethical Considerations [recommended as subheading under "Methods"] (not a CONSORT item)

X26-i) Comment on ethics committee approval

	1	2	3	4	5	
subitem not at all important	○	○	○	○	◉	essential

Clear selection

Does your paper address subitem X26-i?

Copy and paste relevant sections from the manuscript (include quotes in quotation marks "like this" to indicate direct quotes from your manuscript), or elaborate on this item by providing additional information not in the ms, or briefly explain why the item is not applicable/relevant for your study

This study received full board approval from the Human Research Ethics Committee of Khon Kaen University (HE671294). It was also registered with the Thai Clinical Trials Registry prior to participant enrollment (TCTR20240708012). The study was conducted in accordance with the World Medical Association Declaration of Helsinki and institutional policies.

x26-ii) Outline informed consent procedures

Outline informed consent procedures e.g., if consent was obtained offline or online (how? Checkbox, etc.?), and what information was provided (see 4a-ii). See [6] for some items to be included in informed consent documents.

	1	2	3	4	5	
subitem not at all important	○	○	○	○	◉	essential

Clear selection

Does your paper address subitem X26-ii?

Copy and paste relevant sections from the manuscript (include quotes in quotation marks "like this" to indicate direct quotes from your manuscript), or elaborate on this item by providing additional information not in the ms, or briefly explain why the item is not applicable/relevant for your study

Participants received clear written information about the study objectives, procedures, potential risks, and data management before participating. Participation was voluntary, and they could withdraw at any time without affecting their academic standing. Those who agreed to take part then provided written informed consent.

X26-iii) Safety and security procedures

Safety and security procedures, incl. privacy considerations, and any steps taken to reduce the likelihood or detection of harm (e.g., education and training, availability of a hotline)

	1	2	3	4	5	
subitem not at all important	○	○	○	○	◉	essential

Clear selection

Does your paper address subitem X26-iii?

Copy and paste relevant sections from the manuscript (include quotes in quotation marks "like this" to indicate direct quotes from your manuscript), or elaborate on this item by providing additional information not in the ms, or briefly explain why the item is not applicable/relevant for your study

After participating in the study, students created unique codes instead of using their names, which enabled us to link pre- and posttest data without knowing individual identities to protect privacy and confidentiality. Data were anonymized for analysis and stored on password-protected systems with access restricted to the investigator’s team. Students did not receive any financial or in-kind compensation. The manuscript and supplementary materials contain no identifiable images of participants.

RESULTS

13a) For each group, the numbers of participants who were randomly assigned, received intended treatment, and were analysed for the primary outcome

NPT: The number of care providers or centers performing the intervention in each group and the number of patients treated by each care provider in each center

Does your paper address CONSORT subitem 13a? *

Copy and paste relevant sections from the manuscript (include quotes in quotation marks "like this" to indicate direct quotes from your manuscript), or elaborate on this item by providing additional information not in the ms, or briefly explain why the item is not applicable/relevant for your study

A total of 81 participants were assessed for eligibility. Two participants declined to participate. Both were in clusters randomized to the F2F group and therefore received F2F teaching as part of the regular course. However, their data were not included in the analysis. Seventy-nine participants were enrolled in the study and allocated into two groups: the F2F group (n = 38) and the WBVE group (n = 41). One participant from the WBVE group was excluded due to incomplete data. Seventy-eight participants had complete data in the analysis (Figure 2).

13b) For each group, losses and exclusions after randomisation, together with reasons

Does your paper address CONSORT subitem 13b? (NOTE: Preferably, this is shown in a CONSORT flow diagram) *

Copy and paste relevant sections from the manuscript (include quotes in quotation marks "like this" to indicate direct quotes from your manuscript), or elaborate on this item by providing additional information not in the ms, or briefly explain why the item is not applicable/relevant for your study

Two participants declined to participate. Both were in clusters randomized to the F2F group and therefore received F2F teaching as part of the regular course. However, their data were not included in the analysis. … One participant from the WBVE group was excluded due to incomplete data. Seventy-eight participants had complete data in the analysis (Figure 2).

13b-i) Attrition diagram

Strongly recommended: An attrition diagram (e.g., proportion of participants still logging in or using the intervention/comparator in each group plotted over time, similar to a survival curve) or other figures or tables demonstrating usage/dose/engagement.

	1	2	3	4	5	
subitem not at all important	○	○	○	○	◉	essential

Clear selection

Does your paper address subitem 13b-i?

Copy and paste relevant sections from the manuscript or cite the figure number if applicable (include quotes in quotation marks "like this" to indicate direct quotes from your manuscript), or elaborate on this item by providing additional information not in the ms, or briefly explain why the item is not applicable/relevant for your study

The intervention consisted of a single 130-minute teaching session delivered in a supervised classroom/computer-lab setting. There was no use-phase attrition. All students who attended the assigned WBVE or F2F session remained for the full session, and there were no dropouts or logouts during WBVE use.

14a) Dates defining the periods of recruitment and follow-up

Does your paper address CONSORT subitem 14a? *

Copy and paste relevant sections from the manuscript (include quotes in quotation marks "like this" to indicate direct quotes from your manuscript), or elaborate on this item by providing additional information not in the ms, or briefly explain why the item is not applicable/relevant for your study

August 26, 2024, to January 7, 2025.

14a-i) Indicate if critical “secular events” fell into the study period

Indicate if critical “secular events” fell into the study period, e.g., significant changes in Internet resources available or “changes in computer hardware or Internet delivery resources”

	1	2	3	4	5	
subitem not at all important	○	○	○	○	◉	essential

Clear selection

Does your paper address subitem 14a-i?

Copy and paste relevant sections from the manuscript (include quotes in quotation marks "like this" to indicate direct quotes from your manuscript), or elaborate on this item by providing additional information not in the ms, or briefly explain why the item is not applicable/relevant for your study

The study was conducted from August 26, 2024, to January 7, 2025. There was no long-term follow-up beyond the immediate post-class assessment.

14b) Why the trial ended or was stopped (early)

Does your paper address CONSORT subitem 14b? *

Copy and paste relevant sections from the manuscript (include quotes in quotation marks "like this" to indicate direct quotes from your manuscript), or elaborate on this item by providing additional information not in the ms, or briefly explain why the item is not applicable/relevant for your study

The trial ended as planned when all scheduled cohorts completed the one-session intervention; there was no early stopping.

15) A table showing baseline demographic and clinical characteristics for each group

NPT: When applicable, a description of care providers (case volume, qualification, expertise, etc.) and centers (volume) in each group

Does your paper address CONSORT subitem 15? *

Copy and paste relevant sections from the manuscript (include quotes in quotation marks "like this" to indicate direct quotes from your manuscript), or elaborate on this item by providing additional information not in the ms, or briefly explain why the item is not applicable/relevant for your study

Table 1 shows baseline demographics (gender, age, GPA, WBVE experience) for each group and demonstrates comparability.

15-i) Report demographics associated with digital divide issues

In ehealth trials it is particularly important to report demographics associated with digital divide issues, such as age, education, gender, social-economic status, computer/Internet/ehealth literacy of the participants, if known.

	1	2	3	4	5	
subitem not at all important	○	○	○	○	◉	essential

Clear selection

Does your paper address subitem 15-i? *

Copy and paste relevant sections from the manuscript (include quotes in quotation marks "like this" to indicate direct quotes from your manuscript), or elaborate on this item by providing additional information not in the ms, or briefly explain why the item is not applicable/relevant for your study

Our paper reports several baseline characteristics that are directly relevant to digital divide issues, as shown in Table 1. These variables (age, gender, academic Level, prior WBVE exposure, and comfort with the technology) address key aspects of the potential digital divide within this cohort of undergraduate medical students.

16) For each group, number of participants (denominator) included in each analysis and whether the analysis was by original assigned groups

16-i) Report multiple “denominators” and provide definitions

Report multiple “denominators” and provide definitions: Report N’s (and effect sizes) “across a range of study participation [and use] thresholds” [1], e.g., N exposed, N consented, N used more than x times, N used more than y weeks, N participants “used” the intervention/comparator at specific pre-defined time points of interest (in absolute and relative numbers per group). Always clearly define “use” of the intervention.

	1	2	3	4	5	
subitem not at all important	○	○	○	○	◉	essential

Clear selection

Does your paper address subitem 16-i? *

Copy and paste relevant sections from the manuscript (include quotes in quotation marks "like this" to indicate direct quotes from your manuscript), or elaborate on this item by providing additional information not in the ms, or briefly explain why the item is not applicable/relevant for your study

We reported the denominators for all primary analyses in the Results section and in Tables 1 and 2. Seventy-eight students with complete data were included in the primary and secondary analyses (F2F n=38; WBVE n=40). One participant in the WBVE group was excluded from the analysis due to incomplete data. Proportions were calculated using non-missing denominators, as stated in the Statistical analysis section.

16-ii) Primary analysis should be intent-to-treat

Primary analysis should be intent-to-treat, secondary analyses could include comparing only “users”, with the appropriate caveats that this is no longer a randomized sample (see 18-i).

	1	2	3	4	5	
subitem not at all important	○	○	○	○	◉	essential

Clear selection

Does your paper address subitem 16-ii?

Copy and paste relevant sections from the manuscript (include quotes in quotation marks "like this" to indicate direct quotes from your manuscript), or elaborate on this item by providing additional information not in the ms, or briefly explain why the item is not applicable/relevant for your study

The primary analysis followed the intention-to-treat principle. All randomized participants with complete outcome data were analyzed in their originally allocated groups, as described in the Statistical analysis section.

17a) For each primary and secondary outcome, results for each group, and the estimated effect size and its precision (such as 95% confidence interval)

Does your paper address CONSORT subitem 17a? *

Copy and paste relevant sections from the manuscript (include quotes in quotation marks "like this" to indicate direct quotes from your manuscript), or elaborate on this item by providing additional information not in the ms, or briefly explain why the item is not applicable/relevant for your study

For the primary outcome (post-class knowledge), we reported group means, standard deviations, mean differences, 95% confidence intervals, and P-values in the Abstract and Results, and in Table 2 (F2F 11.24 (1.93) vs WBVE 10.40 (2.62); mean difference 0.88, 95% CI −0.18 to 1.94; P=.12). For secondary outcomes (learner satisfaction items), we reported means, standard deviations, mean differences, 95% CIs, and P-values for each domain in Table 2 (for example, overall course satisfaction mean difference 0.42, 95% CI 0.07–0.77; P=.01).

17a-i) Presentation of process outcomes such as metrics of use and intensity of use

In addition to primary/secondary (clinical) outcomes, the presentation of process outcomes such as metrics of use and intensity of use (dose, exposure) and their operational definitions is critical. This does not only refer to metrics of attrition (13-b) (often a binary variable), but also to more continuous exposure metrics such as “average session length”. These must be accompanied by a technical description how a metric like a “session” is defined (e.g., timeout after idle time) [1] (report under item 6a).

	1	2	3	4	5	
subitem not at all important	○	○	○	○	◉	essential

Clear selection

Does your paper address subitem 17a-i?

Copy and paste relevant sections from the manuscript (include quotes in quotation marks "like this" to indicate direct quotes from your manuscript), or elaborate on this item by providing additional information not in the ms, or briefly explain why the item is not applicable/relevant for your study

All participants attended a single 130-minute TBL session and were expected to complete all ten modules in their allocated format (WBVE or F2F). We did not perform separate analyses by platform use intensity (e.g., number of log-ins, time on platform, or partial completion) because exposure to the intervention was standardized, and we did not define distinct "users" versus "non-users" beyond trial allocation.

17b) For binary outcomes, presentation of both absolute and relative effect sizes is recommended

Does your paper address CONSORT subitem 17b? *

Copy and paste relevant sections from the manuscript (include quotes in quotation marks "like this" to indicate direct quotes from your manuscript), or elaborate on this item by providing additional information not in the ms, or briefly explain why the item is not applicable/relevant for your study

Not applicable. All prespecified outcomes (knowledge scores and learner satisfaction ratings) were continuous.

18) Results of any other analyses performed, including subgroup analyses and adjusted analyses, distinguishing pre-specified from exploratory

Does your paper address CONSORT subitem 18? *

Copy and paste relevant sections from the manuscript (include quotes in quotation marks "like this" to indicate direct quotes from your manuscript), or elaborate on this item by providing additional information not in the ms, or briefly explain why the item is not applicable/relevant for your study

No subgroup or post-hoc exploratory analyses were conducted beyond the prespecified primary and secondary analyses.

18-i) Subgroup analysis of comparing only users

A subgroup analysis of comparing only users is not uncommon in ehealth trials, but if done, it must be stressed that this is a self-selected sample and no longer an unbiased sample from a randomized trial (see 16-iii).

	1	2	3	4	5	
subitem not at all important	○	○	○	○	◉	essential

Clear selection

Does your paper address subitem 18-i?

Copy and paste relevant sections from the manuscript (include quotes in quotation marks "like this" to indicate direct quotes from your manuscript), or elaborate on this item by providing additional information not in the ms, or briefly explain why the item is not applicable/relevant for your study

We did not perform user-only analyses or stratified analyses by WBVE use intensity. All participants with complete outcome data were included in the primary and secondary analyses, in line with the intention-to-treat approach.

19) All important harms or unintended effects in each group

(for specific guidance see CONSORT for harms)

Does your paper address CONSORT subitem 19? *

Copy and paste relevant sections from the manuscript (include quotes in quotation marks "like this" to indicate direct quotes from your manuscript), or elaborate on this item by providing additional information not in the ms, or briefly explain why the item is not applicable/relevant for your study

After participating in the study, students created unique codes instead of using their names, which enabled us to link pre- and posttest data without knowing individual identities to protect privacy and confidentiality. Data were anonymized for analysis and stored on password-protected systems with access restricted to the investigator’s team. Students did not receive any financial or in-kind compensation. The manuscript and supplementary materials contain no identifiable images of participants.

19-i) Include privacy breaches, technical problems

Include privacy breaches, technical problems. This does not only include physical “harm” to participants, but also incidents such as perceived or real privacy breaches [1], technical problems, and other unexpected/unintended incidents. “Unintended effects” also includes unintended positive effects [2].

	1	2	3	4	5	
subitem not at all important	○	○	○	○	◉	essential

Clear selection

Does your paper address subitem 19-i?

Copy and paste relevant sections from the manuscript (include quotes in quotation marks "like this" to indicate direct quotes from your manuscript), or elaborate on this item by providing additional information not in the ms, or briefly explain why the item is not applicable/relevant for your study

After participating in the study, students created unique codes instead of using their names, which enabled us to link pre- and posttest data without knowing individual identities to protect privacy and confidentiality. Data were anonymized for analysis and stored on password-protected systems with access restricted to the investigator’s team. Students did not receive any financial or in-kind compensation. The manuscript and supplementary materials contain no identifiable images of participants.

19-ii) Include qualitative feedback from participants or observations from staff/researchers

Include qualitative feedback from participants or observations from staff/researchers, if available, on strengths and shortcomings of the application, especially if they point to unintended/unexpected effects or uses. This includes (if available) reasons for why people did or did not use the application as intended by the developers.

	1	2	3	4	5	
subitem not at all important	○	○	○	○	●	essential

Clear selection

Does your paper address subitem 19-ii?

Copy and paste relevant sections from the manuscript (include quotes in quotation marks "like this" to indicate direct quotes from your manuscript), or elaborate on this item by providing additional information not in the ms, or briefly explain why the item is not applicable/relevant for your study

We did not formally collect qualitative feedback (e.g., free-text comments) during the trial.

DISCUSSION

22) Interpretation consistent with results, balancing benefits and harms, and considering other relevant evidence

NPT: In addition, take into account the choice of the comparator, lack of or partial blinding, and unequal expertise of care providers or centers in each group

22-i) Restate study questions and summarize the answers suggested by the data, starting with primary outcomes and process outcomes (use)

Restate study questions and summarize the answers suggested by the data, starting with primary outcomes and process outcomes (use).

	1	2	3	4	5	
subitem not at all important	○	○	○	○	◉	essential

Clear selection

Does your paper address subitem 22-i? *

Copy and paste relevant sections from the manuscript (include quotes in quotation marks "like this" to indicate direct quotes from your manuscript), or elaborate on this item by providing additional information not in the ms, or briefly explain why the item is not applicable/relevant for your study

In the Conclusions and Discussion, we interpret that WBVE-delivered TBL produced knowledge gains comparable to F2F delivery, but F2F yielded consistently higher learner satisfaction across multiple domains. We therefore conclude that WBVEs cannot fully replace F2F teaching but can serve as a complementary option when physical space, scheduling, or cohort size constrain in-person instruction, provided that facilitation and user experience are optimized.

22-ii) Highlight unanswered new questions, suggest future research

Highlight unanswered new questions, suggest future research.

	1	2	3	4	5	
subitem not at all important	○	○	○	○	◉	essential

Clear selection

Does your paper address subitem 22-ii?

Copy and paste relevant sections from the manuscript (include quotes in quotation marks "like this" to indicate direct quotes from your manuscript), or elaborate on this item by providing additional information not in the ms, or briefly explain why the item is not applicable/relevant for your study

We highlight that WBVEs may be particularly valuable in settings with limited classroom space, tight schedules, or large cohorts, where they can expand access while preserving comparable knowledge outcomes. At the same time, we emphasize the need for reliable devices, robust technical support, and instructor training to manage additional complexity (e.g., navigation, latency, audio issues). Thus, the potential scalability and flexibility benefits of WBVEs must be balanced against the resource requirements and the risk of lower satisfaction if implementation is suboptimal.

20) Trial limitations, addressing sources of potential bias, imprecision, and, if relevant, multiplicity of analyses

20-i) Typical limitations in ehealth trials

Typical limitations in ehealth trials: Participants in ehealth trials are rarely blinded. Ehealth trials often look at a multiplicity of outcomes, increasing risk for a Type I error. Discuss biases due to non-use of the intervention/usability issues, biases through informed consent procedures, unexpected events.

	1	2	3	4	5	
subitem not at all important	○	○	○	○	◉	essential

Clear selection

Does your paper address subitem 20-i? *

Copy and paste relevant sections from the manuscript (include quotes in quotation marks "like this" to indicate direct quotes from your manuscript), or elaborate on this item by providing additional information not in the ms, or briefly explain why the item is not applicable/relevant for your study

We discuss several factors limiting Generalizability in the Limitations section. This is a single-center study in Thai fifth-year medical students with a relatively small sample size and predominantly novice experience with WBVEs. The intervention was delivered on institutional desktop computers within a secure network, so results may not generalize to learners using personal devices, weaker internet connections, or very different institutional cultures. We also note that cultural expectations for structured, instructor-led learning in Thai medical education may influence satisfaction and differ from those in other regions.

21) Generalisability (external validity, applicability) of the trial findings

NPT: External validity of the trial findings according to the intervention, comparators, patients, and care providers or centers involved in the trial

21-i) Generalizability to other populations

Generalizability to other populations: In particular, discuss generalizability to a general Internet population, outside of a RCT setting, and general patient population, including applicability of the study results for other organizations

	1	2	3	4	5	
subitem not at all important	○	○	○	○	◉	essential

Clear selection

Does your paper address subitem 21-i?

Copy and paste relevant sections from the manuscript (include quotes in quotation marks "like this" to indicate direct quotes from your manuscript), or elaborate on this item by providing additional information not in the ms, or briefly explain why the item is not applicable/relevant for your study

Our manuscript addresses generalizability in the Implications and Limitations sections. For example, we state:
Our findings indicate that WBVEs can yield knowledge gains comparable to those of F2F instruction. WBVEs may be especially valuable in settings with limited classroom space, tight schedules, or large cohorts.

21-ii) Discuss if there were elements in the RCT that would be different in a routine application setting

Discuss if there were elements in the RCT that would be different in a routine application setting (e.g., prompts/reminders, more human involvement, training sessions or other co-interventions) and what impact the omission of these elements could have on use, adoption, or outcomes if the intervention is applied outside of a RCT setting.

	1	2	3	4	5	
subitem not at all important	○	○	○	○	◉	essential

Clear selection

Does your paper address subitem 21-ii?

Copy and paste relevant sections from the manuscript (include quotes in quotation marks "like this" to indicate direct quotes from your manuscript), or elaborate on this item by providing additional information not in the ms, or briefly explain why the item is not applicable/relevant for your study

Elements in F2F group are similar to normal practice.

OTHER INFORMATION

23) Registration number and name of trial registry

Does your paper address CONSORT subitem 23? *

Copy and paste relevant sections from the manuscript (include quotes in quotation marks "like this" to indicate direct quotes from your manuscript), or elaborate on this item by providing additional information not in the ms, or briefly explain why the item is not applicable/relevant for your study

Trial registration: TCTR20240708012 was prospectively registered in the Thai Clinical Trials Registry on July 8, 2024.

24) Where the full trial protocol can be accessed, if available

Does your paper address CONSORT subitem 24? *

Cite a Multimedia Appendix, other reference, or copy and paste relevant sections from the manuscript (include quotes in quotation marks "like this" to indicate direct quotes from your manuscript), or elaborate on this item by providing additional information not in the ms, or briefly explain why the item is not applicable/relevant for your study

Thai Clinical Trials Registry

25) Sources of funding and other support (such as supply of drugs), role of funders

Does your paper address CONSORT subitem 25? *

Copy and paste relevant sections from the manuscript (include quotes in quotation marks "like this" to indicate direct quotes from your manuscript), or elaborate on this item by providing additional information not in the ms, or briefly explain why the item is not applicable/relevant for your study

Funding: This study was granted by the Faculty of Medicine, Khon Kaen University, Thailand (Grant Number IN67086).

X27) Conflicts of Interest (not a CONSORT item)

X27-i) State the relation of the study team towards the system being evaluated

In addition to the usual declaration of interests (financial or otherwise), also state the relation of the study team towards the system being evaluated, i.e., state if the authors/evaluators are distinct from or identical with the developers/sponsors of the intervention.

	1	2	3	4	5	
subitem not at all important	○	○	○	○	◉	essential

Clear selection

Does your paper address subitem X27-i?

Copy and paste relevant sections from the manuscript (include quotes in quotation marks "like this" to indicate direct quotes from your manuscript), or elaborate on this item by providing additional information not in the ms, or briefly explain why the item is not applicable/relevant for your study

Conflicts of Interest: None.

About the CONSORT EHEALTH checklist

As a result of using this checklist, did you make changes in your manuscript? *

- ○ yes, major changes
- ◉ yes, minor changes
- ○ no

What were the most important changes you made as a result of using this checklist?

methodology and discussion

How much time did you spend on going through the checklist INCLUDING making changes in your manuscript *

Four hours to do it............

As a result of using this checklist, do you think your manuscript has improved? *

- (●) yes
- ( ) no
- ( ) Other:

Would you like to become involved in the CONSORT EHEALTH group?

This would involve for example becoming involved in participating in a workshop and writing an "Explanation and Elaboration" document

- (●) yes
- ( ) no
- ( ) Other:

Clear selection

Any other comments or questions on CONSORT EHEALTH

none...........................

STOP - Save this form as PDF before you click submit

To generate a record that you filled in this form, we recommend to generate a PDF of this page (on a Mac, simply select "print" and then select "print as PDF") before you submit it.

When you submit your (revised) paper to JMIR, please upload the PDF as supplementary file.

Don't worry if some text in the textboxes is cut off, as we still have the complete information in our database. Thank you!

Final step: Click submit !

Click submit so we have your answers in our database!

Submit

Clear form

Never submit passwords through Google Forms.

This form was created outside of your domain. - Contact form owner - Terms of Service - Privacy Policy

Does this form look suspicious? Report

Google Forms
